# A synergistic cascade nanoreactor breaks bladder cancer chemoresistance: Synergizing cholesterol metabolism reprogramming with cGAS-STING-mediated metallotherapy

**DOI:** 10.1016/j.mtbio.2026.103537

**Published:** 2026-08-06

**Authors:** Xianchun Fu, Ye Liang, Bin Xu, Chao Huang, Pengfei Zhang, Haitao Niu

**Affiliations:** a Department of Urology, The Affiliated Hospital of Qingdao University, Qingdao, 266003, China; b Department of Hepatobiliary and Pancreatic Surgery, The Affiliated Hospital of Qingdao University, Qingdao, 266003, China; c College of Medicine, Southwest Jiaotong University, Chengdu, Sichuan, 610031, China

**Keywords:** Zeolitic imidazolate framework-67, Cholesterol 25-hydroxylase, Cisplatin, cGAS-STING pathway, Bladder cancer, Drug resistance

## Abstract

Bladder cancer chemoresistance arises from a self-reinforcing vicious cycle of aberrant cholesterol metabolism and immunosuppression, which conventional single-target therapies fail to disrupt. Here, we report a folate-targeted zeolitic imidazolate framework-67 (ZIF67)-based nanoreactor termed CCZF, co-loaded with cholesterol 25-hydroxylase (CH25H) and cisplatin, to orchestrate a metabolism-chemo-immunity cascade with mutually amplifying components. First, the intrinsic catalase (CAT)-like activity of ZIF67 generates oxygen in situ to fuel CH25H-mediated cholesterol depletion that reaches 72% in resistant bladder cancer cells. This process disrupts lipid raft structure and downregulates P-glycoprotein (P-gp), resulting in 97.2% cisplatin retention in resistant cells. Second, enhanced cisplatin cytotoxicity induces nucleocytoplasmic translocation of high-mobility group box 1 (HMGB1), an early molecular signature associated with immunogenic cell death (ICD). Third, Co^2+^ released from degraded ZIF67 activates the cyclic GMP-AMP synthase-stimulator of interferon genes (cGAS-STING) pathway. The CH25H-derived metabolite 25-hydroxycholesterol (25HC) acts as an immunomodulatory oxysterol that works with high-mobility group box 1 (HMGB1) to potentiate toll-like receptor 4 (TLR4)-associated innate immune signaling and remodel the immunosuppressive tumor microenvironment. In MB49R tumor-bearing mice, intratumoral CD8^+^ T cell infiltration increases by 2.3-fold on day 14 after treatment. In preclinical bladder cancer models, this tripartite synergy achieves 95.9% short-term tumor growth suppression with no obvious systemic toxicity under the tested dosage and observation period, providing a promising multifaceted strategy for treating chemo-resistant bladder cancer.

## Graphical abstract

**Figure:**
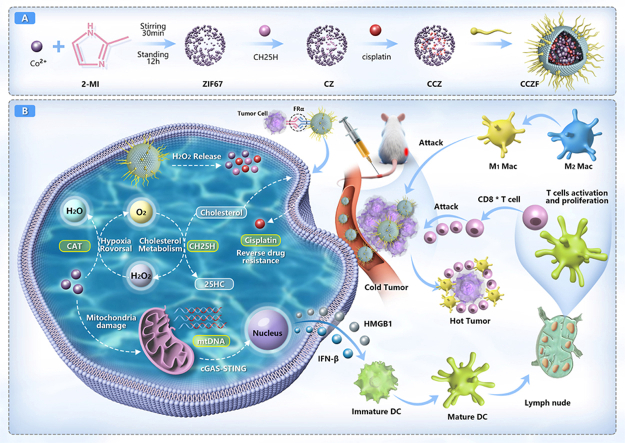

## 1 Introduction

Bladder cancer remains one of the most prevalent malignancies of the urinary tract, and cisplatin-based chemotherapy is the standard first-line treatment for advanced disease [1,2]. Despite initial therapeutic response, the majority of patients eventually develop multidrug resistance (MDR) and suffer disease progression, leading to a dismal 5-year survival rate of less than 30% for metastatic cases [3,4]. Classic mechanisms of chemoresistance have been well documented, including overexpression of drug efflux pumps such as P-glycoprotein (P-gp) [5,6] and enhanced DNA repair activity [7,8]. Recent evidence has identified metabolic reprogramming of the tumor microenvironment (TME) as a more intricate and intractable driver of treatment failure [[9], [10], [11]].

A self-reinforcing vicious cycle drives chemoresistance in bladder cancer. Aberrant cholesterol metabolism [12,13] stabilizes lipid rafts [14,15] to enhance P-gp-mediated drug efflux and direct chemoresistance [16,17], while simultaneously promoting an immunosuppressive TME by accumulating myeloid-derived suppressor cells (MDSCs) and regulatory T cells (Tregs) [18]. Conventional single-target therapies fail catastrophically because they cannot simultaneously disrupt this interconnected network [[19], [20], [21]]. P-gp inhibitors show limited efficacy due to tumor hypoxia [22,23]; Statins cause systemic toxicity and cannot reverse immune suppression [[24], [25], [26]]; Immunotherapies are constrained by the metabolically driven cold TME [[27], [28], [29], [30], [31], [32]].

Nanocarriers co-delivering chemotherapeutics and cyclic GMP-AMP synthase-stimulator of interferon genes (cGAS-STING) agonists have emerged as a promising strategy to combine chemotherapy and immunotherapy [33]. Accumulating evidence from photodynamic immunotherapy also confirms that alleviating tumor hypoxia and metabolic reprogramming can substantially amplify immune activation efficacy, providing a conceptual reference for our cascade design [34]. Unlike simple additive combinations, we developed a folate-targeted zeolitic imidazolate framework-67 (ZIF67)-based nanoreactor named CCZF that integrates three synergistic functionalities into a single platform to break the vicious cycle via a metabolism-chemo-immunity cascade.

We hypothesized that bridging cholesterol metabolism, hypoxia relief, and innate immune activation could synergistically reverse chemoresistance [35]. Cholesterol 25-hydroxylase (CH25H) serves as a potent endogenous effector by converting cholesterol into 25-hydroxycholesterol (25HC), a metabolite that inhibits de novo sterol biosynthesis and disrupts lipid raft integrity [36,37]. Its therapeutic efficacy is however contingent upon sufficient oxygen supply.

Metal-organic frameworks (MOFs), particularly ZIF67, present an ideal carrier to address this limitation [38,39]. ZIF67 exhibits intrinsic catalase (CAT)-like nanozyme activity to decompose endogenous H_2_O_2_ in the tumor microenvironment and generate oxygen in situ. This activity relieves hypoxia limitation and supports CH25H-mediated cholesterol metabolism, theoretically forming a self-sustaining metabolic cascade. ZIF67 also releases cobalt ions (Co^2+^) in the acidic TME [40,41], which triggers mitochondrial damage and subsequent cytosolic mitochondrial DNA leakage to activate the cGAS-STING pathway [[42], [43], [44], [45]].

**Scheme 1:**
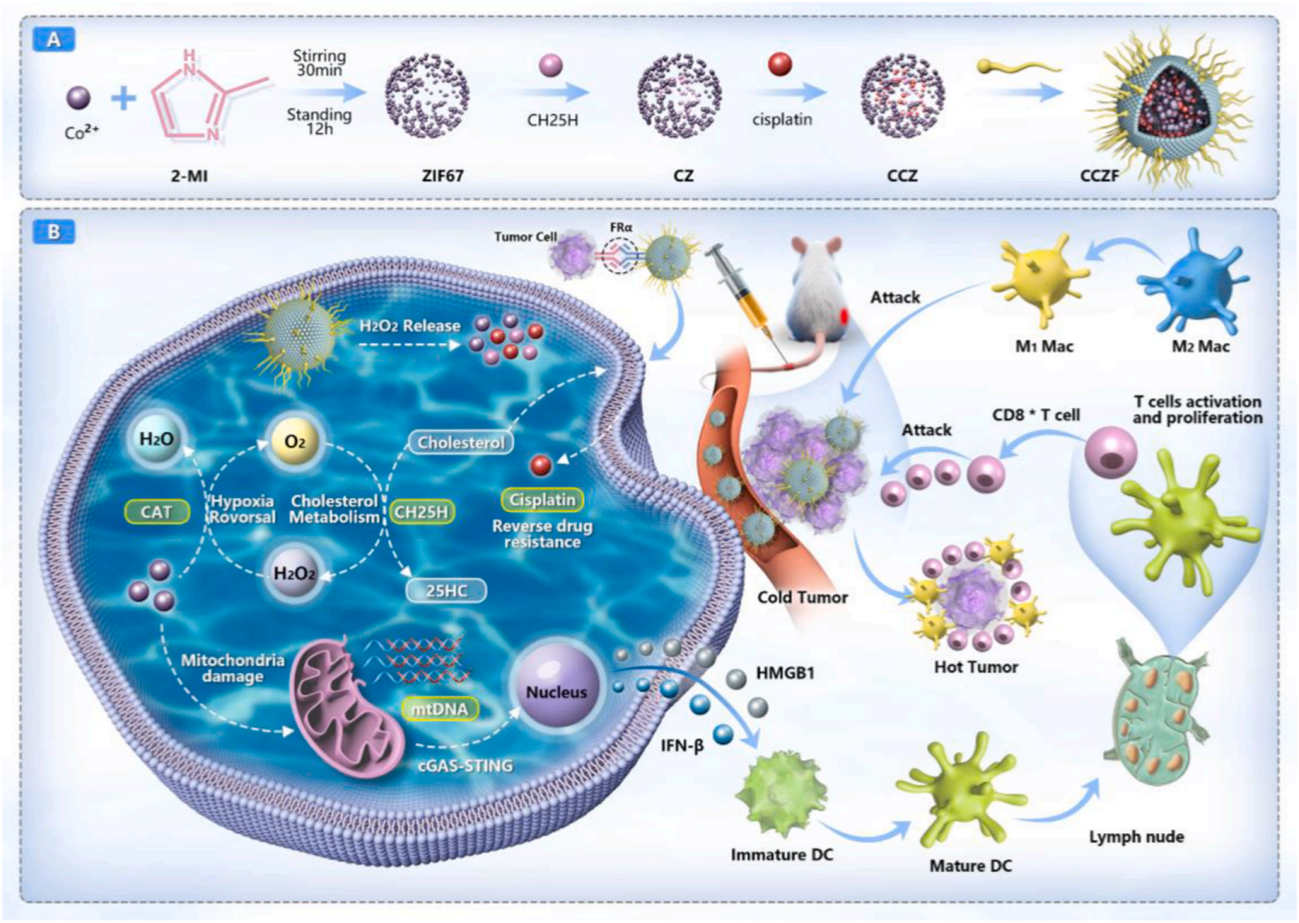
**A**) Schematic illustration of CCZF synthesis. **B**) The metabolism-chemo-immunity cascade for reversing bladder cancer chemoresistance of CCZF.

In this study, we rationally engineered the metabolism-chemo-immunity integrated nanoreactor CCZF, which consists of folate-PEG-modified ZIF67 co-loaded with CH25H and cisplatin. We demonstrate that CCZF establishes a sequential cascade where ZIF67-mediated oxygen generation maximizes CH25H activity, leading to lipid raft collapse and massive cisplatin retention. Simultaneously, cascade release of Co^2+^ and tumor antigens activates STING signaling to transform immune deserts into immune-active tumor sites. This work provides a high-efficacy solution for resistant bladder cancer and offers a reference strategy for treating cholesterol-dependent malignancies through metallo-immunotherapy (Scheme 1).

## 2 Results and discussion

### 2.1 Design and characterization of CCZF

**Fig. 1:**
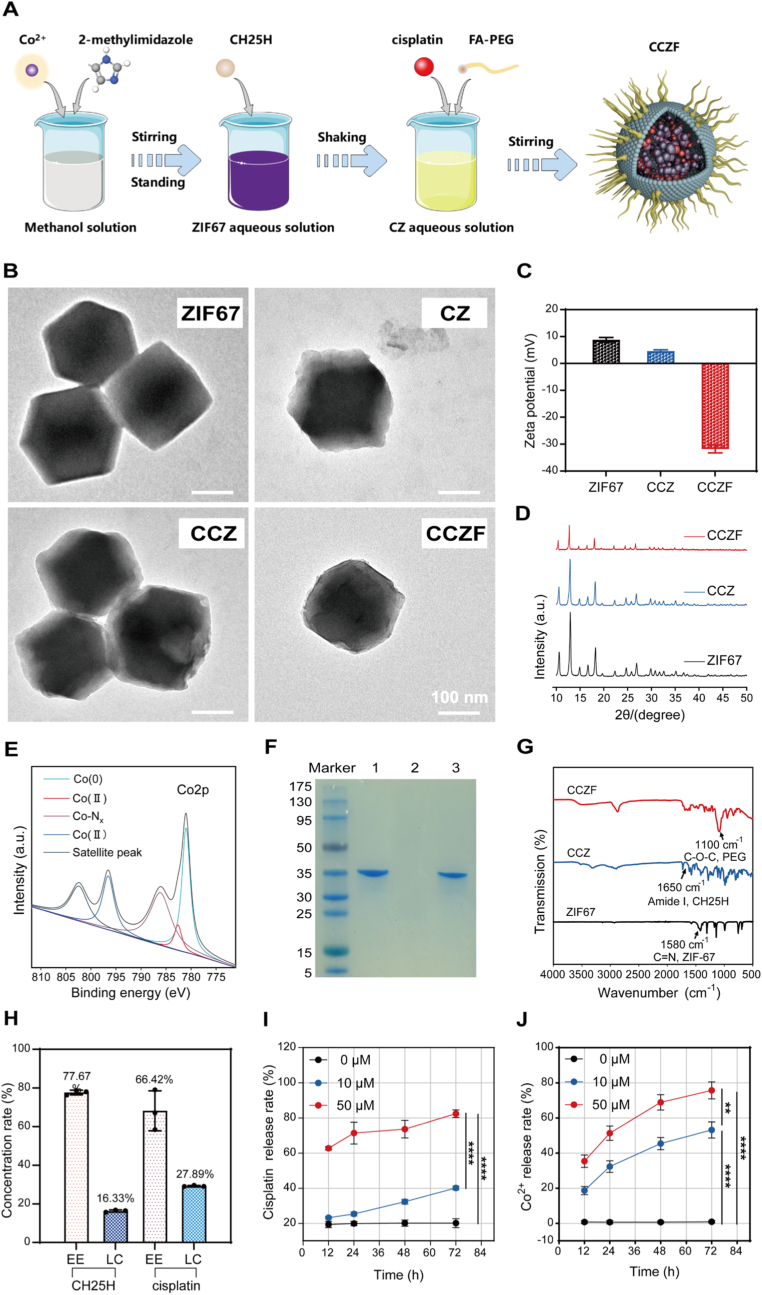
Characterization of CCZF Nanoparticles. A) Schematic diagram of CCZF synthesis route. B) TEM images of ZIF67, CZ, CCZ and CCZF, Scale bar = 100 nm. C) Zeta potential of different formulations. D) XRD patterns of ZIF67, CCZ and CCZF. E) XPS Co 2p spectrum. F) SDS-PAGE for CH25H identification, lane 1 corresponds to CH25H, lane 2 represents ZIF67, and lane 3 denotes CZ. G) FT-IR spectra of ZIF67, CCZ and CCZF nanoparticles. H) Loading and encapsulation efficiency of CH25H and cisplatin. I) H_2_O_2_-mediated catalytic release profiles of Co^2+^ from CCZF. J) H_2_O_2_-mediated catalytic release profiles of cisplatin from CCZF. Statistical analysis was performed by one-way ANOVA with Tukey's post-hoc test. Data are expressed as mean ± SD (n = 3). ns, no significance, *P < 0.05. **P < 0.01, ***P < 0.001, ****P < 0.0001.

To address the unmet need for synergistic metabolism-chemo-immunity modulation in chemo-resistant bladder cancer, we rationally engineered a multifunctional nanoreactor CCZF via a precision-tailored stepwise assembly strategy with detailed protocols provided in Supporting Information. The core design integrates three complementary functionalities within a ZIF67-based core-shell architecture, including targeted co-delivery of CH25H and cisplatin, TME-responsive activation, and preservation of intrinsic biological activity. The surface is further functionalized with folate-polyethylene glycol (FA-PEG) for active tumor targeting. As illustrated in Fig. 1A, the fabrication process was sequentially optimized to ensure seamless functional integration: ZIF67 nanoparticles were first dispersed as a porous, biocompatible scaffold, followed by self-assembled co-loading of CH25H and cisplatin to form the CCZ (CH25H@Cisplatin@ZIF67) intermediate. Final FA-PEG conjugation yielded CCZF (CH25H@Cisplatin@ZIF67@FA-PEG) nanoplatform, which ensures synchronous delivery of the endogenous enzyme and chemotherapeutic agent for coordinated metabolic regulation, chemotherapy, and immune modulation. Control formulations including pristine ZIF67, CZ (CH25H@ZIF67), CisZ (cisplatin@ZIF67), and CCZ were fabricated using identical protocols for mechanistic dissection and comparative analysis (Supporting **Information**).

Comprehensive physicochemical characterization corroborated the successful construction and structural integrity of CCZF across all assembly stages. Transmission electron microscopy imaging (TEM) in Fig. 1B showed that pristine ZIF67 nanoparticles were uniform octahedra with an average diameter of approximately 200 nm. CZ nanoparticles with an average size of 225 nm displayed a distinct surface protein layer, and CCZ nanoparticles of around 255 nm showed increased protein density. Both formulations retained the characteristic octahedral morphology of ZIF67. FA-PEG modification produced CCZF with a size of approximately 260 nm and a smoothed surface, confirming complete functionalization without compromising core architecture. Dynamic light scattering (DLS) further characterized hydrodynamic properties of each formulation, with results shown in Fig. 1C and Fig. S1. ZIF67 exhibited a hydrodynamic diameter of 219.6 ± 18.5 nm with a slightly positive zeta potential of 8.1 ± 0.5 mV. CZ showed a diameter of 239.8 ± 10.5 nm and a potential of 4.6 ± 0.4 mV, and the reduced positivity was consistent with protein coating on the particle surface. Critically, CCZF exhibited a hydrodynamic diameter of 273.3 ± 7.3 nm and a strongly negative zeta potential of −31.3 ± 0.7 mV after modification with carboxyl-terminated FA-PEG. This particle size falls within the optimal window for the enhanced permeability and retention (EPR) effect in solid tumors. The negatively charged PEGylated surface minimizes nonspecific protein adsorption and prolongs blood circulation time. Combined with folate receptor α (FRα)-mediated active targeting, these properties ensure efficient tumor accumulation after intravenous administration. Powder X-ray diffraction (XRD) in Fig. 1D confirmed that the crystalline structure of ZIF67 was well preserved in CCZ and CCZF, with minimal peak shifts indicating negligible structural disruption from cargo loading. X-ray photoelectron spectroscopy (XPS) in Fig. 1E and Fig. S2–S5 identified characteristic Co 2p3/2 and Co 2p1/2 peaks at 781.0 eV and 796.5 eV, verifying predominant Co(Ⅱ) species that are essential for the CAT activity of ZIF67. The high-resolution Pt 4f spectrum exhibited two distinct peaks at 72.4 eV and 75.7 eV, which match well with the characteristic binding energies of Pt(II) in pristine cisplatin [46,47]. This result confirms successful incorporation of cisplatin into the ZIF67 framework and verifies that the chemical structure and valence state of cisplatin remain intact during the encapsulation process. Sodium dodecyl sulfate-polyacrylamide gel electrophoresis (SDS-PAGE) analysis in Fig. 1F confirmed successful CH25H loading in CZ via a prominent band at approximately 35 kDa, which was absent in pristine ZIF67 samples. Fourier transform infrared spectroscopy (FT-IR) was performed to verify the stepwise surface modification of the nanoplatform, with results shown in Fig. 1G. Characteristic peaks of the ZIF67 framework, including C-H stretching at 3130 cm^-1^ and C=N stretching at 1580 cm^-1^, were clearly observed in all formulations. After CH25H loading, CCZ displayed typical amide I (∼1650 cm^-1^) and broad N-H stretching (∼3280 cm^-1^) bands originating from the protein backbone, confirming successful immobilization of CH25H on the ZIF67 scaffold. After FA-PEG conjugation, CCZF showed a distinct C-O-C stretching peak at 1100 cm^-1^, verifying completion of surface functionalization.

Nitrogen adsorption-desorption measurements in Fig. S6–S7 confirmed the highly porous nature of ZIF67 with a Type IV isotherm, H4 hysteresis loop, and a BET surface area = 1669 m^2^ g^-1^. Mesopores centered at 12.7 nm originating from nanoparticle stacking, while the intrinsic microporous structure of the ZIF67 framework was well preserved. These interparticle mesopores provide ample space for CH25H protein and cisplatin loading. Optimization of the cisplatin-to-CZ mass ratio from 1:1 to 1:3 identified the 1:2 ratio as the optimal, as shown in Fig. S8. This ratio yields a CH25H loading capacity of 16.33% with an encapsulation efficiency of 77.67% measured by Bradford assay with Co^2+^ absorbance interference pre-subtracted. It also achieves a cisplatin loading capacity of 27.89% with an encapsulation efficiency of 66.42% measured by inductively coupled plasma mass spectrometry (ICP-MS). Detailed data are presented in Fig. 1H and Fig. S9–S10. This balance of co-delivery efficiency and structural stability is critical for subsequent *in vivo* applications. Scanning transmission electron microscopy coupled with energy-dispersive X-ray spectroscopy (STEM-EDS) elemental mapping analysis was performed to confirm the spatial distribution of cobalt (Co) from the ZIF67 framework and platinum (Pt) from loaded cisplatin. As shown in Fig. S11, the STEM image revealed a well-defined octahedral morphology of CCZF nanoparticles. EDS elemental mapping demonstrated that Co and Pt elements were uniformly distributed throughout the entire nanoparticle without obvious local aggregation or phase separation. This distribution is attributed to coordination-driven diffusion of cisplatin into the ZIF67 framework via Pt-N coordination bonds during the co-incubation loading process. These results directly validate the successful co-loading of cisplatin into the ZIF67 framework and support the TME-responsive synchronous release of Co^2+^ and cisplatin, which is essential for the synergistic cascade of CCZF.

Biosafety and colloidal stability are prerequisites for clinical translation, and both were rigorously validated in this study. CCZF maintained a stable hydrodynamic size and low polydispersity index (PDI) during incubation in phosphate-buffered saline (PBS, pH 7.4) for 7 days and in mouse serum for 24 h, as shown in Figs. S12 and S13. These results indicate favorable colloidal stability under the tested conditions. To further assess its dynamic colloidal behavior under tumor-relevant conditions, we monitored the time-dependent hydrodynamic diameter, PDI, and zeta potential of CCZF over 24 h. Three media conditions were tested, including 10% FBS, pH 6.8 PBS, and pH 6.8 PBS containing 50 μM H_2_O_2_. Results are shown in Figure S14 and Figure S15. CCZF remained largely unchanged in 10% FBS and pH 6.8 PBS, confirming good dispersion stability under serum-containing and mildly acidic conditions. In contrast, CCZF showed progressive increases in particle size and PDI together with a marked shift in zeta potential under H_2_O_2_-containing acidic conditions, indicating oxidative microenvironment-triggered structural transformation. These results further demonstrate that FA-PEG-modified CCZF is sufficiently stable during circulation while retaining responsiveness to the oxidative tumor microenvironment. This property is favorable for site-specific therapeutic activation and controlled payload release. Hemolysis assays performed across a concentration range of 10 to 400 μg mL^-1^ total nanoparticles revealed that CCZF induced less than 4% red blood cell lysis, as shown in Fig. S16. This value is well below the 5% safety threshold for intravenous administration, confirming excellent blood compatibility of CCZF. 3-(4,5-dimethylthiazol-2-yl)-2,5-diphenyltetrazolium bromide (MTT) assays performed on L929 fibroblasts with concentrations up to 400 μg mL^-1^ showed that ZIF67 exerted no significant cytotoxicity at the highest tested concentration, as shown in Fig. S17. Preliminary *in vivo* evaluations confirm minimal carrier-related adverse effects, validating the biocompatibility of ZIF67 as a safe nanocarrier scaffold for therapeutic cargo.

Notably, CCZF exhibits tumor microenvironment-specific H_2_O_2_-responsive cargo release, which is a critical feature for on-target therapeutic activation. As shown in Fig. 1I and Fig. S18, the blue-violet color of ZIF67 faded progressively with increasing H_2_O_2_ concentrations from 100 to 400 μg mL^-1^. Absorbance at 650 nm decreased by 78.3%, which is attributable to H_2_O_2_-mediated oxidation of Co^2+^-imidazolate coordination nodes. This degradation enables synchronous release of CH25H and cisplatin in the TME. Corresponding drug release profiles are presented in Fig. 1J. Under 50 μM H_2_O_2_ to mimic tumor oxidative stress, cumulative cisplatin release reached 68.4% over 72 h, with 24 h and 48 h release rates of 45.2% and 59.7%, respectively. This release level is 3.2-fold higher than that observed under H_2_O_2_-free conditions, which showed 21.3% cumulative release at 72 h. This stimuli-responsive behavior synchronizes cargo release with TME signals, minimizing off-target toxicity while maximizing intratumoral bioavailability of therapeutic agents. To further evaluate release behavior under more clinically relevant conditions, we compared cargo release under typical tumor-mimicking conditions of pH 5.5 with 50 μM H_2_O_2_ against normal physiological conditions of pH 7.4 without H_2_O_2_. Results are shown in Figs. S19 and S20. Both cisplatin and Co^2+^ exhibited markedly accelerated release under acidic conditions with H_2_O_2_, whereas only limited leakage was observed at neutral pH without oxidative stimulus. These results further confirm that CCZF undergoes dual-stimuli-responsive decomposition and controlled payload release specifically in the TME. This pattern favors intratumoral drug activation while reducing premature systemic drug leakage. Notably, acidic pH or H_2_O_2_ alone induced only partial release, while their combination led to the highest cumulative release, indicating a synergistic dual-stimuli-responsive release pattern.

Collectively, these data validate CCZF as a precisely engineered nanoplatform with well-defined structure, stable co-delivery capacity, favorable biosafety, and TME-responsive behavior. By integrating targeted delivery, metabolic regulation, and on-demand drug release, CCZF lays a critical foundation for synergistic therapy in chemo-resistant bladder cancer and addresses key limitations of single-mechanism therapies. The stepwise assembly strategy ensures synchronous delivery of all three functional components, which is a prerequisite for the proposed metabolism-chemo-immunity triad.

### 2.2 CAT-like activities of CCZF: establishing the synergistic cascade metabolic foundation

**Fig. 2:**
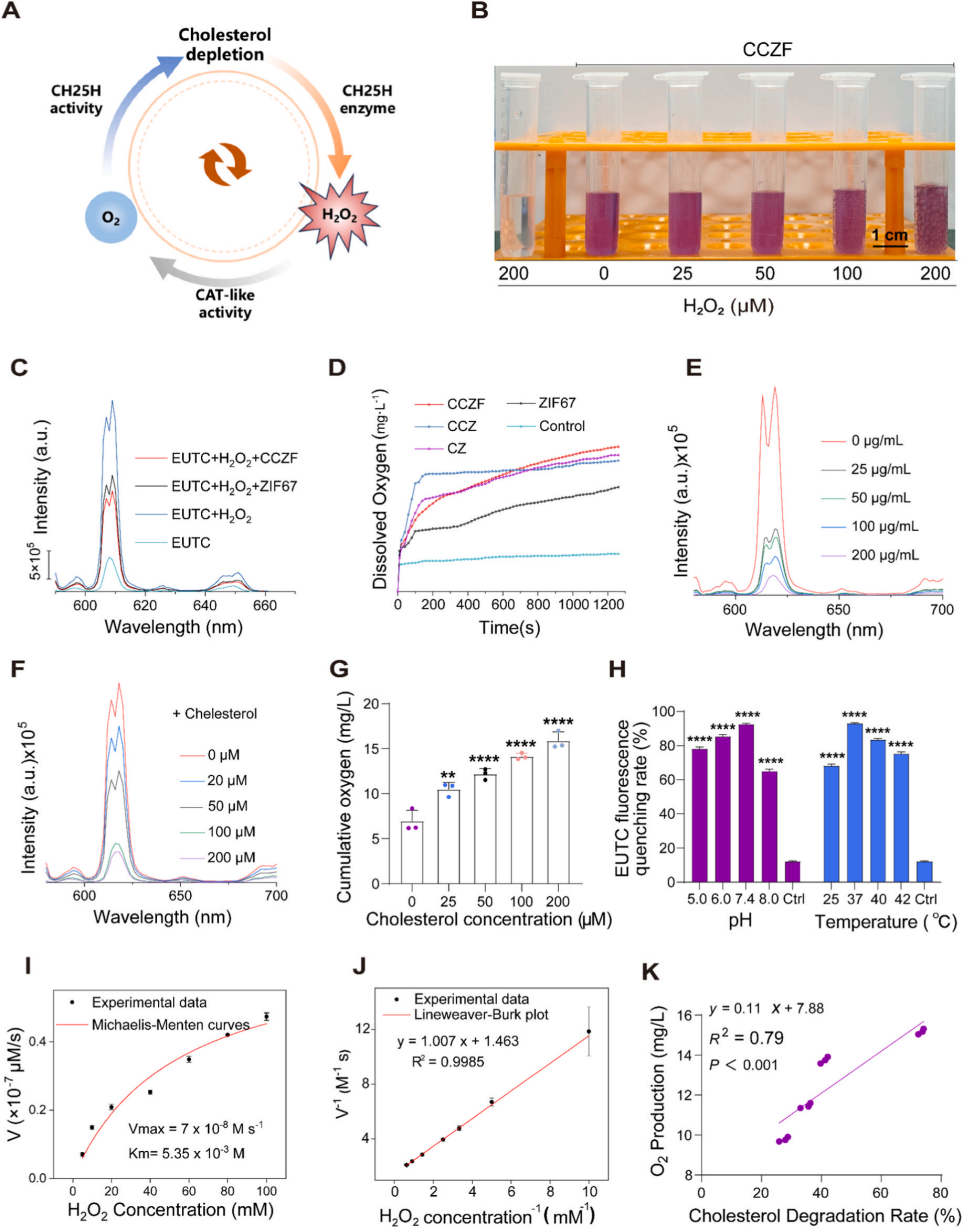
*In vitro* catalytic properties of CCZF. A) Schematic of self-sustained O_2_-CH25H metabolic cycle. B) Optical photos of O_2_ bubble generation under gradient H_2_O_2_, Scale bar = 1 cm. C) EUTC fluorescence detecting residual H_2_O_2_. D) Dissolved oxygen dynamic curves. E) Dose-dependent CAT activity of CCZF. F) Cholesterol gradient dependent catalytic performance. G) Quantitative cumulative oxygen production at varied cholesterol concentrations. H) pH and temperature influence on catalytic activity. I/J) Michaelis-Menten and Lineweaver-Burk fitting for CCZF. K) Correlation between cholesterol degradation and O_2_ generation. All quantitative data: n = 3 and were analyzed by one-way ANOVA with Tukey post-test. Data shown as mean ± SD. ns, no significance, *P < 0.05. **P < 0.01, ***P < 0.001, ****P < 0.0001.

The synergistic crosstalk between ZIF67's CAT-like nanozyme activity and CH25H's native enzymatic function forms the core therapeutic innovation of the CCZF system. To elucidate this cooperative interplay, we propose a self-sustaining metabolic cascade hypothesis. ZIF67 decomposes endogenous H_2_O_2_ derived from tumor oxidative stress into molecular O_2_ via its intrinsic CAT-like activity, and the in situ generated O_2_ in turn fuels CH25H-mediated hydroxylation of excess membrane cholesterol into 25HC. This tandem catalytic arrangement amplifies cholesterol metabolism suppression and preserves CH25H enzymatic activity even in the hypoxic TME. Mechanistically, ZIF67-mediated oxygen generation relieves hypoxia-induced inhibition of CH25H activity. We hypothesize that sustained cholesterol metabolic reprogramming may in turn maintain tumor cellular oxidative stress and a continuous H_2_O_2_ supply, which theoretically forms a functionally self-reinforcing metabolic loop that remains to be directly validated in cellular contexts. This upstream metabolic cascade alleviates hypoxia in the acidic TME, an issue that has long been a critical bottleneck for oxygen-dependent enzymatic therapies. It also drives continuous cholesterol depletion, thereby disrupting lipid raft stability to reverse cholesterol-mediated chemoresistance (Fig. 2A).

To rigorously validate this synergistic paradigm, we first interrogated ZIF67's CAT-like activity using europium-tetracycline (EUTC) fluorescent probes, which emit characteristic fluorescence at 620 nm upon binding to H_2_O_2_. As shown in Fig. S21, ZIF67 nanoparticles induced a 45% reduction in fluorescence intensity, directly demonstrating efficient H_2_O_2_ decomposition. Extending this assay to the full CCZF platform, results in Fig. 2C showed that the H_2_O_2_-alone group exhibited maximal fluorescence set as 100%, while the H_2_O_2_ + ZIF67 and H_2_O_2_ + CCZF groups displayed significant quenching with relative intensities of 55.2 ± 3.1% and 52.8 ± 2.7%, respectively. No statistically significant difference was observed between ZIF67 and CCZF, confirming that FA-PEG modification does not impair the catalytic activity of ZIF67. This preserved activity is essential for in situ O_2_ generation in the hypoxic TME. Visual validation of oxygen bubble formation further corroborated the intrinsic catalase-like activity of the ZIF67-based nanoplatform. Negligible bubbles were observed in the 0 μM H_2_O_2_ group, while bubble quantity and density increased progressively with H_2_O_2_ concentration and peaked at the highest tested dose, as shown in Fig. 2B. This result provides direct visual evidence that ZIF67 mediates dose-dependent H_2_O_2_ decomposition and O_2_ generation. The robust in situ oxygen production capacity lays the upstream catalytic foundation for the tandem metabolic cascade: O_2_ generated by ZIF67 continuously fuels CH25H-mediated cholesterol hydroxylation, which matches the substrate supply-demand coupling required for the hypothesized self-sustaining metabolic cycle. Quantitative dissolved oxygen measurements were recorded at 20-s intervals, and obvious differences in O_2_ evolution were observed between groups, as shown in Fig. S22. The mixture of ZIF67 and H_2_O_2_ exhibited rapid and continuous oxygen production, with dissolved oxygen levels rising steadily throughout the test. By contrast, the H_2_O_2_-only control group showed negligible spontaneous decomposition, with dissolved oxygen levels remaining nearly unchanged throughout the 60 s observation period. To quantify O_2_ generation dynamics, we monitored dissolved oxygen levels over 20 min in five groups (control, H_2_O_2_ + ZIF67, H_2_O_2_ + CZ, H_2_O_2_ + CCZ, H_2_O_2_ + CCZF) in pH 6.8 PBS at 37°C. As shown in Fig. 2D, the control group exhibited negligible O_2_ changes, while ZIF67, CZ, CCZ, and CCZF groups showed increased dissolved oxygen levels. CCZF displayed the highest peak and most sustained oxygen release among all formulations. No significant difference between ZIF67 and CZ confirmed that CH25H loading does not impair O_2_ production. These results underscore the superior capacity of CCZF to sustain O_2_ generation, which is critical for overcoming hypoxic TME and supporting continuous cholesterol oxidation.

Complementary EUTC fluorescence assays further delineated the catalytic properties of CCZF. At a fixed 10-min reaction time, fluorescence intensity decreased in a CCZF concentration-dependent manner, confirming dose-tunable catalytic activity as shown in Fig. 2E. Under constant CCZF concentration, fluorescence intensity dropped rapidly within 3 min and then stabilized from 4 to 30 min, as shown in Fig. S23. This pattern indicates immediate activation and sustained catalytic stability of the nanoplatform. To explore the CH25H-ZIF67 cascade in the absence of exogenous H_2_O_2_, we analyzed reactions across gradient cholesterol concentrations with results shown in Fig. 2F. Results showed CH25H enzymatically hydroxylated cholesterol to generate 25HC, while H_2_O_2_ was decomposed by ZIF67 to produce O_2_ for sustaining the enzymatic reaction. This process led to measurable fluorescence quenching and directly validated the tandem catalytic cascade between CH25H and ZIF67. Quantitative confirmation of cholesterol-dependent O_2_ generation was obtained by incubating CCZF with gradient cholesterol concentrations in pH 6.8 PBS containing 50 μM H_2_O_2_ at 37°C for 30 min. As shown in Fig. 2G, the cumulative oxygen production increased progressively from 8.3 ± 0.1 mg L^-1^ at 0 μM cholesterol to 15.2 ± 0.2 mg L^-1^ at 200 μM cholesterol. All cholesterol-treated groups showed statistical significance versus the 0 μM control group. The cholesterol-dependent elevation in cumulative oxygen production can be explained by the coupled cascade mechanism. Higher cholesterol concentrations enhance the metabolic flux of CH25H and continuously consume dissolved oxygen, which shifts the catalytic equilibrium of ZIF67 toward more H_2_O_2_ decomposition and results in higher total oxygen output over the incubation period. The net increase in dissolved oxygen indicates that the oxygen generation rate of ZIF67 exceeds the oxygen consumption rate of CH25H, ensuring sufficient oxygen supply for the enzymatic reaction. The slowing growth trend at high cholesterol concentrations aligns with typical enzyme saturation kinetics, further validating that oxygen generation is tightly coupled to cholesterol metabolism in the CCZF-mediated cascade.

To evaluate CCZF's adaptability to physiological and TME conditions, we assessed its CAT-like activity under gradient pH (5.0, 6.0, 7.4, 8.0) and temperature (25°C, 37°C, 40°C, 42°C) using EUTC fluorescence quenching rate as the readout. CCZF retained substantial CAT-like activity (65.0%) under pH 5.0, which mimics the acidic tumor microenvironment, although its optimal activity was observed at pH 7.4 (92.5 ± 0.6%) (Fig. 2H). For temperature dependence, activity peaked at 37°C (92.8 ± 0.7%) and remained substantial at 40°C (83.5 ± 0.7%) and 42°C (75.2 ± 1.1%), while being lower at 25°C (68.2 ± 1.1%). All CCZF-treated groups showed significant differences versus the blank control (****P < 0.0001), confirming robust activity under acidic TME (pH 5.0) and physiological conditions (pH 7.4, 37°C). This property is a key prerequisite for *in vivo* applicability of the nanoplatform. Additionally, CCZF exhibited negligible peroxidase (POD)-like activity (Fig. S24), indicating that it catalyzes the decomposition of H_2_O_2_ into O_2_ rather than cytotoxic hydroxyl radicals (·OH). This property minimizes off-target oxidative damage to normal tissues. To confirm the synergistic coupling between cholesterol degradation and O_2_ generation, we simultaneously quantified cholesterol degradation rate, 25HC accumulation and O_2_ production across gradient cholesterol concentrations. As shown in Fig. 2K, a strong positive correlation was observed between cholesterol degradation and 25HC accumulation (R^2^ = 0.87, *P <* 0.001), while O_2_ production increased proportionally to cholesterol degradation (R^2^ = 0.79, *P <* 0.001). These results directly validate that CCZF-mediated O_2_ generation and 25HC accumulation are tightly coupled to cholesterol metabolic flux, which is consistent with the synergistic framework of the hypothesized self-sustaining metabolic cascade. Kinetic studies using Michaelis-Menten curves further quantified the CAT-like activity of CCZF. The reaction rate increased progressively with H_2_O_2_ concentrations from 0.1 to 1.6 mM, showing typical enzyme-like saturation kinetics as shown in Fig. 2I. A Lineweaver-Burk plot (Fig. 2J) revealed a linear relationship between 1/[S] and 1/v, confirming Michaelis-Menten behavior and providing quantitative insights into CCZF's affinity for H_2_O_2_ (Km = 5.35 × 10^-3^ M) and maximal catalytic rate (Vmax), which further validates the efficient catalytic performance of CCZF.

To directly assess whether the encapsulation process compromised CH25H function, the enzymatic activity of free and CCZF-encapsulated CH25H was measured at a fixed cholesterol concentration. As shown in Fig. S25, CCZF-encapsulated CH25H retained 91.1% of the activity of free CH25H, indicating that the loading process caused only minor enzymatic loss. Negligible signal was detected in the blank CCZF control. These results confirm that CH25H largely preserves its catalytic activity after incorporation into the MOF framework. The enzymatic activity of CH25H under hypoxic conditions (1% O_2_, 5% CO_2_, 94% N_2_, 37°C) was further examined to evaluate its functional stability in a tumor-relevant microenvironment. As shown in Fig. S26, free CH25H exhibited a reduction in activity under hypoxia and retained 78.8% of its normoxic level. In contrast, CCZF-encapsulated CH25H maintained 93.8% of its normoxic activity under the same hypoxic conditions. These findings suggest that the CCZF framework helps preserve CH25H catalytic function under hypoxia stress.

To quantitatively assess whether MOF encapsulation affects the catalytic behavior of CH25H, enzyme kinetic analysis was performed using cholesterol as the substrate. Initial reaction velocities of free CH25H and CCZF-encapsulated CH25H were measured over a range of substrate concentrations and fitted using the Michaelis–Menten model (Fig. S27, Supporting Information). As summarized in Table S1, free CH25H exhibited a Vmax of 105.2 ± 4.3 nmol min^-1^·mg^-1^ and a Km of 48.6 ± 3.1 μM, whereas CCZF-encapsulated CH25H showed a Vmax of 95.8 ± 3.9 nmol min^-1^·mg^-1^ and a Km of 61.4 ± 4.5 μM. The encapsulated enzyme retained 91.1% of the Vmax of free CH25H, indicating that most of the catalytic activity was preserved after incorporation into the MOF structure. The slightly elevated Km value of CCZF-encapsulated CH25H suggests reduced apparent substrate affinity, which may be attributed to diffusion limitations or steric effects within the porous framework. The modest decrease in Vmax indicates that the maximum catalytic turnover was only mildly affected by encapsulation. These results collectively demonstrate that the CCZF platform largely maintains CH25H activity while introducing only limited kinetic hindrance. Corresponding Lineweaver–Burk plots are provided in Fig. S28, Supporting Information.

To further validate the proposed O_2_-CH25H synergistic cascade, we next examined whether CCZF could elevate tumor oxygenation *in vivo* and functionally preserve CH25H-mediated cholesterol oxidation under hypoxic conditions. Real-time intratumoral oxygen monitoring was performed in MB49R tumor-bearing mice using an oxygen microsensor after administration of PBS, free CH25H, CZ, or CCZF. As shown in Fig. S29, the PBS and free CH25H groups exhibited negligible changes in tumor oxygen content throughout the monitoring period and remained close to the hypoxic baseline. In contrast, CZ treatment moderately increased intratumoral O_2_ levels, whereas CCZF induced a much more rapid, pronounced, and sustained oxygenation response. Oxygen content rose to approximately 17 mmHg at 20 min and remained significantly elevated over 60 min. These results provide direct *in vivo* evidence that CCZF can generate O_2_ in situ within the hypoxic TME.

To assess whether ZIF67-derived oxygen could sustain CH25H activity under oxygen limitation, we measured cholesterol degradation under both normoxic conditions (21% O_2_, 5% CO_2_, 37°C) and hypoxic conditions (1% O_2_, 5% CO_2_, 37°C). As shown in Fig. S30, hypoxia markedly reduced the cholesterol-degrading activity of free CH25H, whereas CZ and CCZF retained substantially higher activity, with CCZF showing the strongest cholesterol depletion. Based on these data, the relative activity retention under hypoxia was calculated (Fig. S31), showing that free CH25H retained only 36.90% of its normoxic activity, compared with 67.44% for CZ and 83.54% for CCZF. In the absence of exogenous H_2_O_2_, the activity retention of encapsulated CH25H is mainly attributed to the conformational protection provided by the MOF scaffold. When H_2_O_2_ is present to simulate the tumor oxidative microenvironment, in situ oxygen generation by ZIF67 further rescues CH25H activity under hypoxic conditions, confirming the dual benefits of scaffold protection and oxygen supply. These findings directly support the proposed O_2_-CH25H synergistic cascade.

Collectively, these findings establish CCZF's synergistic cascade mechanism as a paradigm of rational nanoplatform design. ZIF67's CAT-like nanozyme activity and CH25H's natural enzymatic function act in a tandem catalytic cascade to achieve in situ oxygen regeneration and sustained cholesterol depletion. They overcome two interconnected barriers to bladder cancer therapy, hypoxia and cholesterol-mediated chemoresistance. This mechanistic validation lays a critical foundation for CCZF's translational potential and highlights its capacity to advance precision therapy for chemo-resistant bladder cancer. Based on the coupled relationship between cholesterol metabolism and oxygen consumption, we propose that enhanced cholesterol metabolic reprogramming may maintain tumor oxidative stress to sustain H_2_O_2_ supply. This supply in turn drives continuous oxygen production by ZIF67 and forms a self-sustaining metabolic cycle as a testable mechanistic hypothesis. This metabolic foundation serves as the upstream driver of both subsequent chemo-sensitization and immune modulation.

### 2.3 Metabolic remodeling directly mediates chemo-sensitization in resistant bladder cancer cells

**Fig. 3:**
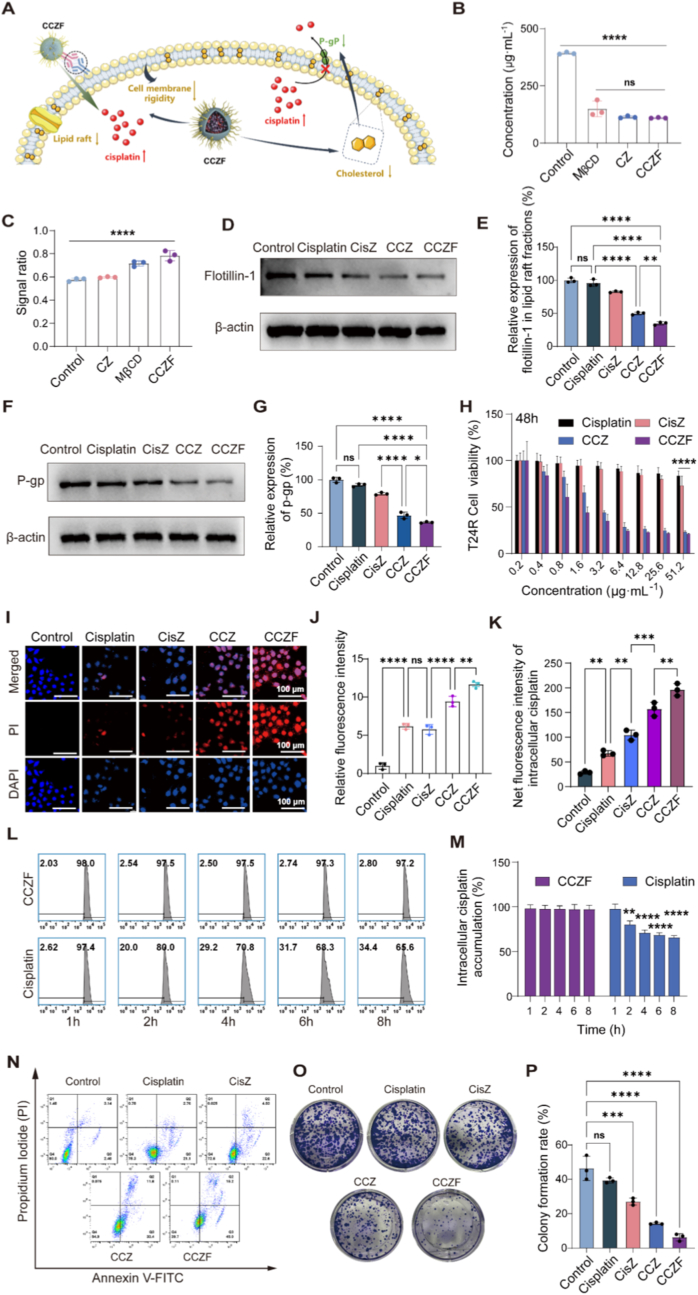
*In vitro* anti-tumor effects against resistant T24R cells. A) Schematic of cholesterol-mediated drug resistance mechanism. B) Intracellular cholesterol content (n = 3). C) Cell membrane fluidity detection (n = 3). D, E) Western blot and quantification of Flotillin-1 (n = 3). F, G) Western blot and quantification of P-gp (n = 3). H) Cell viability of T24R after 48 h treatment (n = 4). I) Confocal immunofluorescence of intracellular cisplatin, Scale bar = 100 μm. J, K) Quantitative cisplatin uptake (n = 3, one-way ANOVA). L, M) Flow cytometry-based cisplatin efflux and retention kinetics (n = 3). N) Apoptosis flow analysis (n = 4). O, P) Colony formation photos and statistical quantification (n = 4, Scale bar = 1 cm for colony plate). All statistical comparisons were performed by one-way ANOVA with Tukey's post-test; ns, no significance, *P < 0.05. **P < 0.01, ***P < 0.001, ****P < 0.0001, data shown as mean ± SD. For nanoparticle formulation groups, concentrations are normalized to equivalent cisplatin content for direct comparison with free cisplatin.

Excessive cholesterol accumulation is a defining hallmark of multidrug resistance (MDR) in bladder cancer. It rigidifies cell membranes, stabilizes lipid raft structures, and upregulates drug efflux pumps to impair chemotherapy efficacy. This section systematically validates the causal cascade linking CCZF-mediated cholesterol depletion to enhanced chemosensitivity in T24R cells. The sequential mechanism: cholesterol depletion → increased membrane fluidity → lipid raft disruption → P-gp downregulation → enhanced cisplatin retention → increased apoptosis → 10.4-fold chemo-sensitization, as illustrated in Fig. 3A. Prior to mechanistic experiments, the cisplatin-resistant phenotype of T24R cells was validated. The 48 h IC_50_ of cisplatin in T24R cells was 201.4 μg mL^-1^, significantly higher than 19.4 μg mL^-1^ in parental T24 cells, corresponding to a resistance index of 10.4-fold (Fig. S38–S40). The resistance index is consistent with established MDR bladder cancer models. T24R cells also exhibited elevated intracellular cholesterol levels and upregulated expression of cisplatin resistance-related proteins, consistent with the cholesterol-dependent chemo-resistant phenotype. To quantify the metabolic remodeling effect of CCZF, we first measured intracellular cholesterol levels in T24R cells after treatment. Untreated T24R cells exhibited a high baseline cholesterol concentration of 392.2 ± 4.6 μM, consistent with the cholesterol-addicted phenotype of chemo-resistant tumors. In contrast, CCZF treatment (50 μg mL^-1^, 24 h) reduced intracellular cholesterol in T24R cells by 72% to 109.8 ± 1.8 nmol mg^-1^ protein (*P* < 0.0001) (Fig. 3B). Methyl-β-cyclodextrin (MβCD, 5 mM, 2 h treatment), a well-established rapid cholesterol-depleting agent, was included as a mechanistic positive control to validate the causal link between cholesterol reduction and membrane fluidity elevation. No direct potency comparison was performed between MβCD and CCZF due to the different action kinetics and treatment durations. This cholesterol depletion was both time- and dose-dependent and reached a plateau at 24 h (Fig. S32). Notably, the concentration of 25-hydroxycholesterol (25HC), the direct product of CH25H-mediated cholesterol hydroxylation, increased proportionally with CCZF concentration. It peaked at 212.5 ± 8.9 μM in the 50 μg mL^-1^ CCZF group at 24 h (Fig. S33). These results confirm that CCZF efficiently drives cholesterol hydroxylation metabolism and inhibits de novo sterol biosynthesis, achieving dual-targeted cholesterol depletion in resistant cells. The reduction in intracellular cholesterol directly translated to enhanced cell membrane fluidity. Membrane fluidity analysis using 1-pyrenedecanoic acid as a fluorescent probe showed that CCZF treatment significantly increased the membrane fluidity of T24R cells from 0.58 ± 0.01 (untreated control) to 0.78 ± 0.05 (*P* < 0.0001) (Fig. 3C). This effect was comparable to that of MβCD treatment, confirming that the increased membrane fluidity is a direct consequence of CCZF-mediated cholesterol depletion.

Increased membrane fluidity disrupts the integrity of lipid rafts, which are specialized membrane microdomains that serve as platforms for drug efflux pumps function. Western blot analysis of purified lipid raft fractions revealed that CCZF treatment significantly reduced Flotillin-1 abundance in lipid raft fractions, a well-established lipid raft marker, by 38.2% ± 3.7% compared to untreated controls (Fig. 3D and E). In contrast, free cisplatin and CisZ (Cisplatin@ZIF67) had minimal effects on Flotillin-1 levels in raft fractions (95.3% ± 4.8% and 83.7% ± 4.5% of control levels, respectively), while CCZ (CH25H@Cisplatin@ZIF67) showed an intermediate effect (52.6% ± 4.1%). These results suggest disruption of lipid raft structural integrity, and CH25H-mediated cholesterol depletion contributes to this process. Lipid rafts are critical for the stability and functional activity of the drug efflux pump P-gp, a key mediator of MDR in T24R cells. Consistent with the lipid raft disruption observed above, CCZF treatment significantly downregulated P-gp expression in membrane proteins by 37.8% ± 3.5% (Fig. 3F and G). Again, free cisplatin and CisZ had negligible effects on P-gp expression (93.6% ± 4.8% and 82.3% ± 3.9% of control levels, respectively), while CCZ showed a partial reduction (51.7% ± 4.2%). To definitively confirm that P-gp downregulation is caused by cholesterol depletion rather than off-target effects, we performed a cholesterol replenishment experiment. Exogenous cholesterol supplementation partially restored P-gp expression in CCZF-treated cells (Fig. S34–S35), while cholesterol treatment alone at the same concentration had no significant effect on P-gp levels compared to controls. This provides direct causal evidence that CCZF-induced cholesterol depletion is necessary and sufficient for P-gp downregulation.

The downregulation of P-gp directly translated to enhanced intracellular cisplatin retention in T24R cells. Confocal microscopy revealed that CCZF-treated cells exhibited the most intense nuclear cisplatin fluorescence, indicating efficient cellular uptake and nuclear localization (Fig. 3I). Quantitative fluorescence intensity analysis showed that CCZF increased intracellular cisplatin accumulation by 1.9-fold compared to free cisplatin (Fig. 3J and K). Most importantly, flow cytometry-based cisplatin efflux kinetics demonstrated that CCZF significantly elevated intracellular cisplatin retention and sustained intracellular platinum accumulation in T24R cells. Free cisplatin was progressively expelled from cells over time, with only 65.6% remaining after 8 h, while CCZF (10 μg mL^-1^ cisplatin equivalent) maintained over 97.2% intracellular cisplatin retention relative to the 0 h time point (immediately after drug loading) in T24R cells throughout the entire 8 h observation period (Fig. 3L and M). This exceptional retention was significantly higher than that of CisZ (78.3%) and CCZ (89.5%). Cholesterol replenishment experiments further confirmed that this enhanced cisplatin retention is dependent on cholesterol depletion. Exogenous cholesterol supplementation (100 μM, 24 h pretreatment) partially reversed the effect of CCZF and restored cisplatin efflux capacity (Fig. S36–S37). These results collectively indicate that CCZF markedly improves intracellular cisplatin retention through multiple contributing mechanisms. Enhanced intracellular cisplatin retention conferred potent cytotoxicity against T24R resistant cells. MTT assays showed that CCZF reduced T24R cell viability to 21.0% at 48 h, with an IC_50_ of 19.42 μg mL^-1^ calculated as cisplatin-equivalent concentration (Fig. S38), representing a 10.4-fold increase in chemosensitivity compared with free cisplatin (IC_50_ = 201.4 μg mL^-1^, Fig. S39). The cisplatin-sensitive phenotype of parental T24 cells was verified in parallel (Fig. S40). Comparative viability analysis across formulations revealed that CCZF exerted the strongest growth inhibition in both T24 (Fig. S41–S42) and T24R cells (Figure S43 and Fig. 3H), with significance reaching *P* < 0.0001 in T24R cells at 24 and 48 h. As shown in Fig. 3N and Fig. S44, the control group had a basal apoptosis rate of 4.7%. Free cisplatin induced 23.3% total apoptosis, which rose moderately to 27.7% in the CisZ group. The CCZ and CCZF groups further increased total apoptosis to 45.3% and 60.3%, respectively. Colony formation assays confirmed the long-term proliferative inhibition effect of CCZF, with the CCZF group exhibiting the lowest colony formation rate (19.8% ± 3.2%), significantly lower than CCZ (38.5% ± 4.1%), CisZ (67.3% ± 5.8%), and free cisplatin (70.5% ± 6.1%) groups (Fig. 3O and P).

Importantly, CCZF exhibited excellent biocompatibility with normal human bladder epithelial SV-HUC-1 cells. MTT assay showed that CCZF treatment resulted in 97.2% ± 4.3% cell viability, which was not significantly different from the control group (100.0% ± 4.2%, ns, P = 0.317) (Fig. S45). In contrast, free cisplatin caused significant cytotoxicity to normal cells, reducing viability to 66.5% ± 5.1% (*P* < 0.0001 vs Control). Collectively, these results establish the first arm of our metabolism-chemo-immunity triad: metabolic remodeling directly mediates potent chemo-sensitization. The sequential cascade of 72% cholesterol depletion → increased membrane fluidity → 38.2% Flotillin-1 downregulation → 37.8% P-gp downregulation → 97.2% cisplatin retention → 60.3% apoptosis → 10.4-fold chemo-sensitization provides definitive evidence that CCZF overcomes cholesterol-mediated MDR by targeting the root cause of drug resistance rather than just inhibiting individual efflux pumps.

### 2.4 Combined metabolic-chemotherapeutic effects synergistically activate antitumor immunity via three convergent pathways

**Fig. 4:**
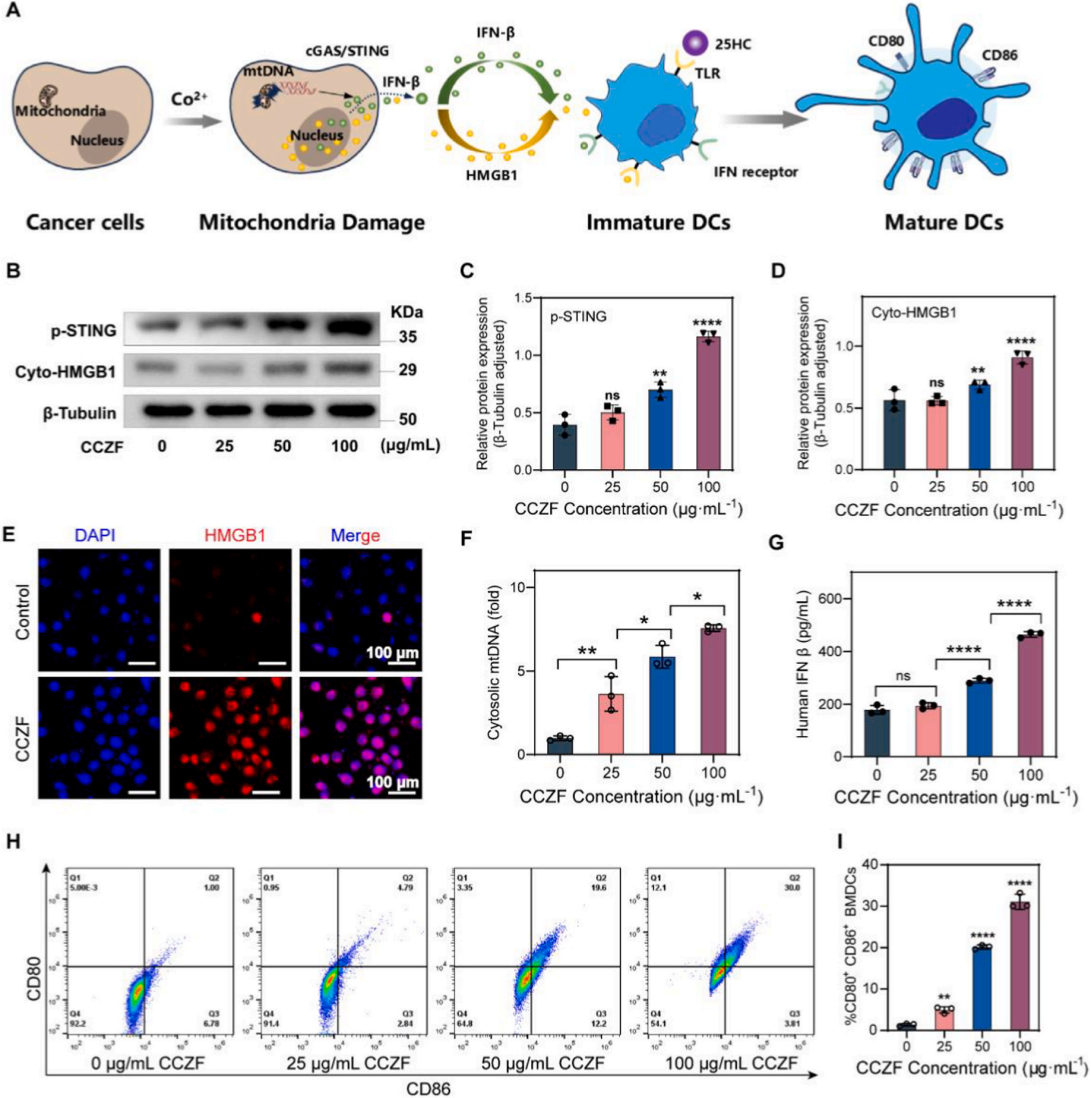
CCZF triggers mtDNA leakage, STING activation and DC maturation. A) Schematic of immune activation cascade. B-D) Western blot and relative quantitative expression of p-STING and cytoplasmic HMGB1 (n = 3). E) HMGB1 cellular translocation immunofluorescence, Scale bar = 100 μm. F) Cytosolic mtDNA quantification (n = 3). G) IFN-β secretion by ELISA (n = 3). H, I) Flow cytometry of mature CD80^+^ CD86^+^ BMDC and statistical analysis (n = 3). Statistics were performed by one-way ANOVA with Tukey post-test. Data are presented as mean ± SD, ns, no significance, *P < 0.05. **P < 0.01, ***P < 0.001, ****P < 0.0001.

Immune activation by CCZF is not solely dependent on the cGAS-STING pathway but is a synergistic effect of three convergent pathways that collectively drive robust antitumor immunity (Fig. 4A). The first pathway is Co^2+^-mediated cGAS-STING activation. H_2_O_2_-triggered degradation of the ZIF67 framework releases Co^2+^ ions that induce mitochondrial damage and subsequent mitochondrial DNA (mtDNA) leakage into the cytoplasm [48,49]. This leads to dose-dependent phosphorylation of STING (p-STING) and interferon-β (IFN-β) secretion (Fig. 4B–C, F-G). The second pathway is cisplatin-associated immunogenic stimulation. Metabolic remodeling-augmented cisplatin cytotoxicity drives nucleocytoplasmic translocation of HMGB1, which is an early molecular signature associated with immunogenic cell death (ICD) and a key damage-associated molecular pattern (DAMP) that primes innate immune responses (Fig. 4B–D-E). Metal-based therapeutic agents have been widely reported to induce potent ICD and remodel the tumor immune microenvironment for cancer immunotherapy [50]. The third pathway is 25HC-mediated immunomodulatory synergy. CH25H-mediated cholesterol metabolism produces 25HC, an immunomodulatory oxysterol that cooperates with HMGB1 to potentiate toll-like receptor 4 (TLR4) signaling in dendritic cells (DC). This effect amplifies IFN-β secretion and DC maturation and establishes a metabolic-immune regulatory link between cholesterol remodeling and innate immune activation. To dissect the temporal dynamics and relative contribution of each pathway, we examined the expression of key molecular markers at 6, 12, and 24 h after CCZF treatment, and additionally pretreated T24R cells with the selective STING inhibitor H-151 (1 μM) for 2 h before CCZF exposure (Fig. S46–S51). The results showed that cytoplasmic mtDNA levels began to increase at 6 h and peaked at 12 h, with the 50 μg mL^-1^ CCZF group exhibiting a 4.2-fold increase compared with the control group. Notably, the IFN-β concentration in the 50 μg mL^-1^ CCZF group (386.7 ± 12.5 pg mL^-1^) was significantly higher than that in the 25 μg mL^-1^ group (218.3 ± 9.6 pg mL^-1^, *P* < 0.0001). As shown in Figs. S49 and S50, CCZF treatment significantly increased the phosphorylation levels of STING, TBK1, and IRF3, and these effects were markedly attenuated by H-151 pretreatment.

We next examined the role of high mobility group box 1 (HMGB1), a well-characterized DAMP that mediates DC maturation [51]. Western blot analysis revealed that CCZF treatment induced a concentration-dependent accumulation of HMGB1 in the cytoplasm (Fig. 4B and D). Confocal microscopy further confirmed this translocation, showing reduced nuclear localization of HMGB1 and concomitant increased cytoplasmic distribution in CCZF-treated T24R cells (Fig. 4E). Quantitative analysis of total HMGB1 fluorescence intensity showed a significant increase in CCZF-treated cells compared with untreated controls (*P* < 0.0001, Fig. S52). These findings collectively confirm that CCZF induces nucleocytoplasmic translocation of HMGB1 in T24R cells. Notably, HMGB1 can synergize with cGAS-STING pathway activation by binding to TLR4 on DCs, thereby amplifying IFN-β secretion and DC maturation [52]. This further supports the role of HMGB1 in modulating the tumor immune microenvironment. Together with the increased IFN-β secretion, these results indicate that CCZF promotes DC maturation through the coordinated release of HMGB1, IFN-β, and 25HC. To evaluate the functional consequences of these molecular events on DC maturation, we assessed the immunomodulatory effects of conditioned medium from CCZF-treated T24R cells on bone marrow-derived dendritic cells (BMDCs). BMDCs were isolated from the femurs and tibias of C57BL/6 mice according to an established protocol [53] (Fig. S53, Supporting Information) and cultured with conditioned medium from T24R cells for 48 h. Flow cytometric analysis revealed that conditioned medium from CCZF-treated T24R cells significantly increased the percentage of CD80^+^ CD86^+^ mature BMDCs compared with control groups (Fig. 4H and I). This effect may result from the combined actions of tumor-derived DAMPs (HMGB1), CH25H-derived 25HC, and released cobalt ions from the degraded nanoplatform. The relative contribution of each component and potential direct effects of residual drugs on DCs require further dissection.

These results collectively reveal a cascade of immunostimulatory events initiated by CCZF. First, Co^2+^-mediated mitochondrial damage and mtDNA leakage activate the cGAS-STING pathway to stimulate IFN-β production. Second, CH25H-derived 25HC acts as an immunomodulatory metabolite that cooperates with HMGB1 to potentiate TLR4-driven IFN-β secretion in DCs. Third, HMGB1 nucleocytoplasmic translocation synergizes with 25HC and IFN-β to potently induce DC maturation. Various STING-activating nanoplatforms have been developed to boost antitumor immunity in recent studies [54]. This cascade lays the foundation for remodeling the immunosuppressive TME. These results establish the second and third arms of our triad: metabolic and chemotherapeutic effects synergize to drive immune activation. This is in stark contrast to single-mechanism immunotherapies that only target one pathway.

To explore the molecular mechanism of the treatment, we performed RNA-seq analysis with three biological replicates per group. The overall distribution of differentially expressed genes (DEGs) is presented in the volcano plot (Fig. S54). We labeled several key genes closely related to cholesterol metabolism and innate immune response. We further visualized the expression patterns of these candidate genes via a row-wise Z-score normalized heatmap (Fig. S55), which showed that cholesterol synthesis-related genes were markedly downregulated, whereas genes involved in cholesterol efflux and immune activation were significantly upregulated. Cluster analysis divided these genes into two independent expression modules, indicating a correlation between metabolic reprogramming and immune activation at the transcriptomic level, while the causal regulatory relationship remains to be established. To clarify the biological functions of DEGs, we conducted GO and KEGG pathway enrichment analyses. The results showed that upregulated genes were mainly enriched in immune response, inflammatory response and type I interferon signaling pathways (Fig. S56), while downregulated genes were primarily associated with cholesterol metabolism and lipid biosynthesis (Fig. S57). Consistent with GO results, KEGG enrichment analysis further confirmed that upregulated genes were concentrated in multiple innate immune signaling pathways and downregulated genes were enriched in lipid and cholesterol metabolic pathways (Figs. S58 and S59). Taken together, transcriptomic data demonstrated that the treatment simultaneously inhibited cholesterol metabolism and activated immune-related pathways.

### 2.5 *In vivo* validation of the tripartite synergy: superior antitumor efficacy and TME remodeling

**Fig. 5:**
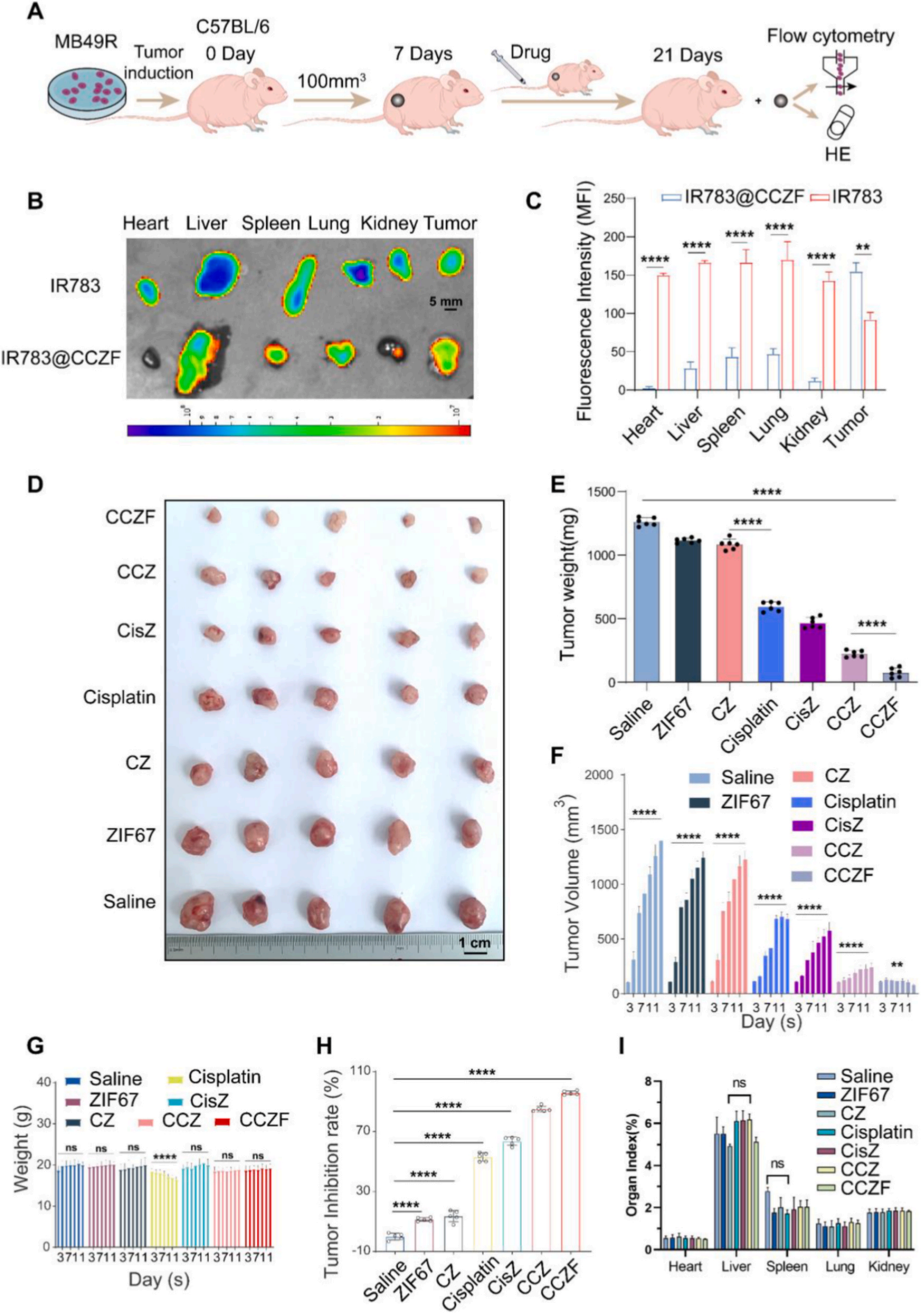
*In vivo* tumor accumulation and therapeutic efficacy in MB49R-bearing C57BL/6 mice (n = 5 per group). A) *In vivo* animal experimental timeline. B) *Ex-vivo* fluorescence images of major organs and tumors at 24 h post-injection. Fluorescence imaging is for qualitative demonstration of tumor accumulation only. Accurate quantification of intact nanoparticles is based on ICP-MS elemental analysis. Scale bar = 5 mm. C) Fluorescence quantitative statistics (n = 5). D) Excised tumor digital photos, Scale bar = 1 cm. E) Average tumor weight (n = 5). F) Tumor volume growth curves (n = 5). G) Body weight variation (n = 5). H) Tumor inhibition rate (n = 5). I) Major organ coefficient statistics (n = 5). Statistical analysis via one-way ANOVA followed by Tukey test. ns, no significance, *P < 0.05. **P < 0.01, ***P < 0.001, ****P < 0.0001. Data presented as mean ± SD.

To further evaluate the *in vivo* antitumor and immunomodulatory effects of CCZF in an immunocompetent host, we established an MB49R syngeneic bladder tumor model in C57BL/6 mice, with the experimental design outlined in Fig. 5A. We first assessed the *in vivo* biodistribution of nanoparticles by intravenously administering IR783-labeled CCZF or free IR783 to tumor-bearing mice. *Ex vivo* fluorescence imaging at 24 h post-injection revealed that CCZF nanoparticles exhibited significantly higher tumor accumulation compared with free IR783 (Fig. 5B and C), demonstrating the enhanced permeability and retention (EPR) effect of the nanoplatform. It should be noted that IR783-based fluorescence imaging only provides semi-qualitative information on tissue distribution, as partial premature leakage of the physically loaded dye may occur *in vivo*, leading to overestimated signals in kidneys and lungs. The contribution of folate receptor-mediated active targeting to tumor accumulation was further validated by a free folic acid competition assay (Fig. S60). To accurately quantify the biodistribution of intact nanoreactors, we further determined cobalt content in major organs via ICP-MS as a more reliable elemental tracer. The results confirmed that intact CCZF particles were mainly enriched in tumor tissue, liver and spleen due to reticuloendothelial system uptake. Only minimal content was detected in lungs and kidneys, consistent with the typical biodistribution pattern of negatively charged nanoparticles of this size range. The gradual decrease of cobalt levels in all organs and detectable cobalt excretion in urine further verified the favorable metabolic clearance profile of CCZF (Fig. S61–S62). Pharmacokinetic analysis further confirmed the favorable *in vivo* profile of CCZF. It achieved a 3.8-fold higher maximum cisplatin concentration in tumor tissues (708.7 ± 32.6 ng g^-1^ vs 186.5 ± 15.3 ng g^-1^ for free cisplatin) and a 4.3-fold higher tumor AUC_0-24_ h (11648.1 vs 2685.5 ng h g^-1^), providing the pharmacological basis for its potent antitumor efficacy (Fig. S63, Table S2). To dissect the synergistic effects *in vivo*, we compared formulations with graded functional components. Single-function intervention included CZ for metabolic regulation only and free cisplatin for chemotherapy only. The dual-function combination was CisZ, which combines chemotherapy and cobalt-mediated immune activation. The triple-function combination without active targeting was CCZ, integrating metabolism, chemotherapy and immune activation. The final formulation was CCZF, the triple-function combination with folate targeting. Post-treatment analysis of excised tumors revealed striking differences in therapeutic efficacy across the seven groups. The CCZF group achieved the most superior outcomes, with the lowest average tumor weight, smallest tumor volume, and an impressive tumor inhibition rate of 95.87% (Fig. 5D–F, H). In contrast, free cisplatin treatment resulted in significantly larger tumors (595.0 ± 36.5 mg) with only 53.09% inhibition, confirming the limited efficacy of conventional chemotherapy against drug-resistant bladder cancer. The superior efficacy of CCZ over CisZ directly demonstrated the synergistic effect of metabolic reprogramming combined with chemo-immunotherapy. The further enhanced efficacy of CCZF compared with CCZ was attributed to folate receptor-mediated active targeting, which increased intratumoral drug accumulation and amplified the triple-mechanism synergy. The triple combination achieved superior efficacy than any single or dual intervention. Longitudinal tumor growth curves further demonstrated that only CCZF nanoparticles induced significant tumor regression, whereas all other groups showed progressive tumor growth throughout the monitoring period (Fig. 5F).

**Fig. 6:**
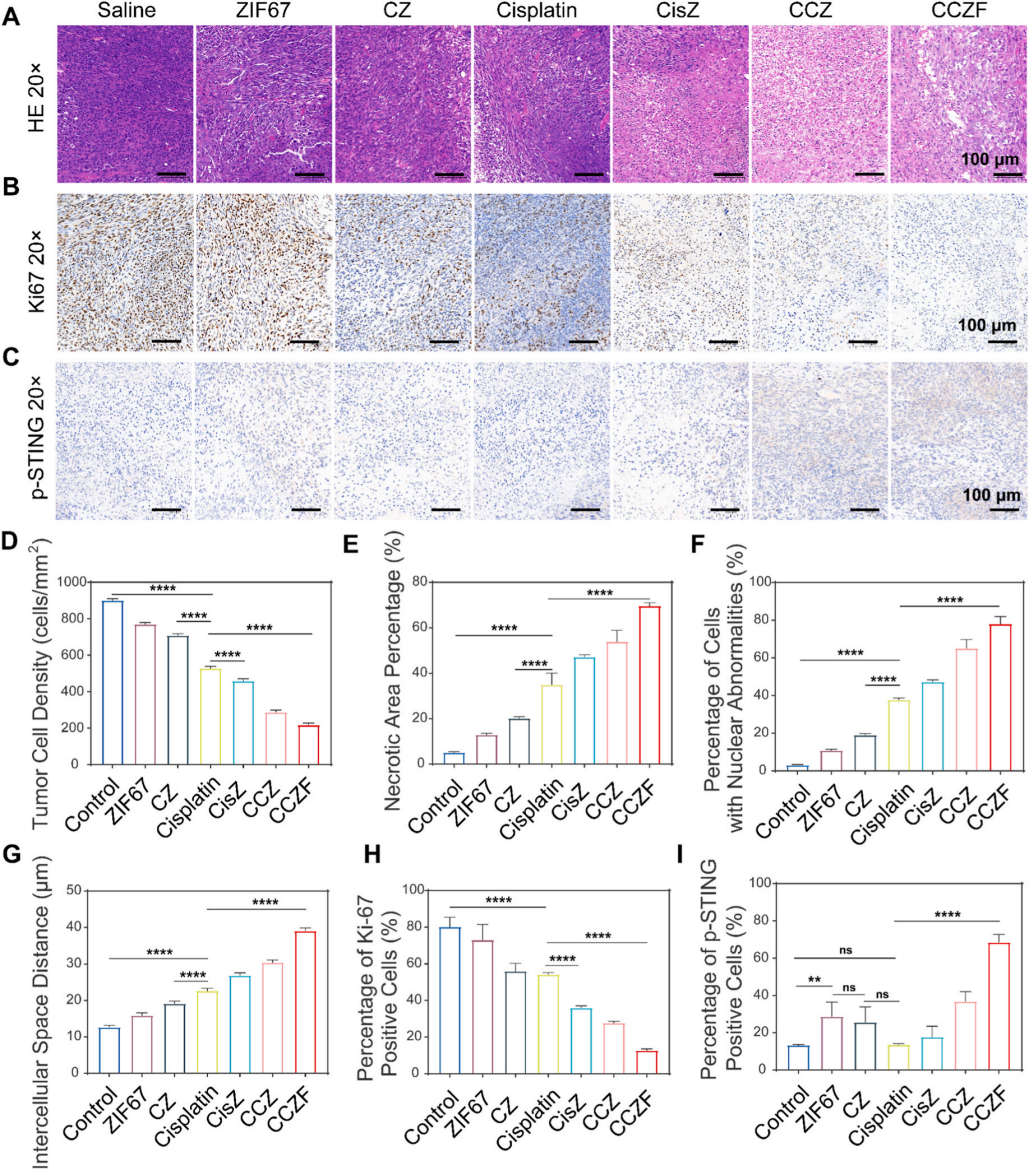
Tumor histopathology and IHC staining after treatment. A) H&E staining of tumor slices; B) Ki-67 IHC; C) p-STING IHC; Scale bar = 100 μm. D-G) Quantification of cell density, necrosis ratio, nuclear damage, intercellular space (n = 5). H) Ki-67 positive cell percentage (n = 5). I) p-STING positive cell percentage (n = 5). Statistical method: one-way ANOVA with Tukey post-hoc test. ns, no significance, *P < 0.05. **P < 0.01, ***P < 0.001, ****P < 0.0001. Data are presented as mean ± SD.

Comprehensive histopathological and immunohistochemical analyses of excised tumor tissues further elucidated the *in vivo* antitumor mechanisms. H&E staining revealed that the CCZF group exhibited the most severe pathological damage, with the lowest tumor cell density, largest necrotic area, highest percentage of cells with nuclear condensation and fragmentation, and most significant widening of intercellular spaces (Fig. 6A–D–G). Consistent with these findings, the CCZF-treated group had a significantly lower percentage of Ki-67-positive cells compared with all other groups (Fig. 6B–H), directly confirming that CCZF effectively suppresses tumor cell proliferation. Most strikingly, the CCZF group exhibited the highest percentage of p-STING-positive cells (Fig. 6C–I), providing clear *in vivo* evidence of enhanced cGAS-STING pathway activation, which is a critical molecular signature contributing to its potent antitumor effects.

Flow cytometric analysis revealed that CCZF significantly remodeled the tumor immune microenvironment compared with both the saline control and free cisplatin groups. The gating strategy for identifying all immune cell subsets in tumor tissues and tumor-draining lymph nodes (TDLNs) is detailed in Fig. S64. Cells were sequentially gated to exclude debris, discriminate singlets, and select live CD45^+^ leukocytes, followed by subset-specific marker gating for CD8^+^ T cells, Tregs, MDSCs, M2 macrophages, and mature DCs. This gating protocol was consistently applied across all treatment groups. In TDLNs, CCZF treatment significantly increased the percentage of mature DCs (CD11c^+^ MHC II^+^), whereas free cisplatin had only minimal effects on DC maturation (Figs. S65 and S66), indicating that CCZF more effectively enhances antigen presentation and subsequent T-cell priming. Consistently, CCZF also significantly increased the infiltration of CD8^+^ cytotoxic T cells into tumor tissues by 2.3-fold, to a much greater extent than free cisplatin (Figs. S67 and S68). In addition to promoting innate and adaptive immune activation, CCZF significantly alleviated immunosuppression within the tumor microenvironment. It reduced the percentage of M2-like macrophages to 17.6 ± 2.8%, whereas free cisplatin increased M2 macrophage polarization to 43.7 ± 4.2%. It also significantly decreased the percentages of Gr-1^+^ CD11b^+^ MDSCs and CD4^+^ Foxp3^+^ Tregs (Fig. S69–S74). Time-course analysis showed that these immune remodeling effects were sustained. In the CCZF group, the percentages of M2 macrophages, MDSCs, and Tregs decreased progressively from day 3 to day 14, while the percentage of CD8^+^ T cells increased continuously and reached 27.6% on day 14 (Fig. S75). Importantly, the degree of TME remodeling was positively correlated with tumor inhibition rate, indicating that immune activation is a critical contributor to the superior efficacy of CCZF.

Body weight changes and comprehensive safety evaluations provided critical insights into the systemic safety of the different formulations. Mice treated with free cisplatin experienced progressive weight loss, reflecting systemic toxicity and tissue damage, whereas all nanoparticle-treated groups maintained stable body weight and normal growth patterns (Fig. 5G). Organ coefficient measurements showed only minor changes in spleen weight in the cisplatin group, whereas all nanoparticle-treated groups maintained normal organ indices (Fig. 5I). Histopathological examination of major organs (heart, liver, spleen, lungs, kidneys) by H&E staining showed preserved tissue architecture and no obvious pathological changes in CCZF-treated mice (Fig. S76), confirming minimal systemic toxicity. Blood biochemical analysis further revealed that free cisplatin significantly elevated liver enzymes (ALT, AST) and renal function markers (CREA, UREA) compared with saline controls, whereas CCZF-treated mice maintained normal hepatic and renal function parameters (Fig. S77). To further address potential long-term biosafety concerns associated with cobalt-containing nanoplatforms, we conducted an extended systemic toxicity evaluation for 30 days after the completion of treatment. During this period, CCZF-treated mice maintained stable body weight and showed no obvious behavioral abnormalities, and serum biochemical analysis and histopathological examination revealed no significant abnormalities in liver and kidney function or tissue structure (Fig. S78–S80). Quantitative determination of residual cobalt levels by ICP-MS showed that cobalt was mainly distributed in the liver and spleen at early time points and gradually declined over time, with cobalt detected in urine samples confirming progressive systemic clearance rather than long-term tissue retention (Fig. S61–S62). These *in vivo* results collectively validate the metabolism-chemo-immunity triad. CCZF simultaneously achieves metabolic remodeling, chemo-sensitization, and immune activation in tumor tissue, leading to potent therapeutic efficacy against chemo-resistant bladder cancer while maintaining favorable systemic tolerance and no obvious toxicity within the observation period.

### 2.6 Study limitations

This work comprehensively demonstrates the synergistic correlations among cholesterol metabolic reprogramming, cisplatin chemo-sensitization, and innate immune activation triggered by the CCZF nanoreactor in chemo-resistant bladder cancer models. Nevertheless, several limitations remain to be addressed in future studies. First, the precise molecular mechanism by which cholesterol metabolism reprogramming reverses cisplatin resistance requires further validation. P-glycoprotein downregulation may be a concomitant event rather than a direct mediator, and potential contributions from platinum transporters (e.g., ATP7A/B, MRP2) and DNA repair pathways need to be systematically clarified. Second, the catalytic efficiency of the ZIF67 nanozyme under physiological intratumoral H_2_O_2_ concentrations may be lower than that measured in cell-free systems, and the actual *in vivo* catalytic flux remains to be quantitatively determined. Third, the relative contributions of folate-mediated active targeting and triple-mechanism synergy to *in vivo* antitumor efficacy have not been fully decoupled. Fourth, only colloidal size stability was evaluated in serum incubation assays; potential premature payload leakage in the systemic circulation was not systematically assessed. Finally, all current conclusions are based on murine tumor cell line-derived xenograft models; validation in patient-derived tumor models and human primary cells is required to support clinical translation.

## 3 Conclusion

This work establishes a metabolism-chemo-immunity triad that disrupts the self-reinforcing vicious cycle of metabolic dysregulation, chemoresistance, and immunosuppression underlying chemo-resistant bladder cancer. In contrast to conventional additive combination therapies that act on individual pathways independently, the CCZF nanoreactor integrates three synergistic functional modules into a single platform, wherein each component reciprocally amplifies the effects of the others to yield superior therapeutic efficacy. Metabolic reprogramming mediates chemo-sensitization via a proposed self-sustaining O_2_-CH25H metabolic cascade. ZIF67-mediated in situ oxygen generation alleviates the long-standing bottleneck of hypoxia-restricted enzymatic therapy and sustains CH25H-driven cholesterol depletion. This process disrupts lipid raft integrity and downregulates P-glycoprotein (P-gp) expression, resulting in a 10.4-fold increase in cisplatin sensitivity in chemoresistant bladder cancer cells. The combined effects of metabolic remodeling and chemotherapy in turn elicit robust antitumor immunity via three convergent mechanisms. First, Co^2+^ released from degraded ZIF67 activates the cyclic GMP-AMP synthase-stimulator of interferon genes (cGAS-STING) pathway. Second, augmented cisplatin cytotoxicity triggers nucleocytoplasmic translocation of HMGB1, an early molecular hallmark of immunogenic cell death (ICD). Third, CH25H-derived 25-hydroxycholesterol (25HC) functions as an immunomodulatory oxysterol that synergizes with HMGB1 to potentiate toll-like receptor 4 (TLR4)-mediated innate immune signaling. Collectively, these events convert immunologically “cold” tumors into “hot” ones—a transformation unattainable by any single pathway alone. This tripartite synergy accounts for the 95.9% tumor growth suppression rate observed in preclinical bladder cancer models, a value markedly superior to that of any single-mechanism or dual-mechanism regimen assessed in this work. Importantly, this strategy reverses chemoresistance by targeting the interconnected regulatory network of metabolism and immunity, rather than merely inhibiting individual drug efflux pumps or immune checkpoints. CCZF also exhibits favorable systemic biocompatibility, with no discernible adverse effects observed within the study observation period, supporting its potential for further translational investigation. Beyond its demonstrated efficacy against chemo-resistant bladder cancer, the modular core-shell architecture of CCZF offers a versatile platform for further optimization. This strategy bears translational potential for other cholesterol-dependent malignancies and may be combined with immune checkpoint inhibitors to further amplify systemic antitumor immunity, providing a reference paradigm for metallo-immunotherapy in cholesterol-dependent refractory cancers.

## 4 Experimental section

### 4.1 Synthesis of CCZF nanoparticles

ZIF67 nanoparticles were first synthesized via the reaction of cobalt nitrate hexahydrate with 2-methylimidazole in methanol solution at room temperature. Subsequently, CH25H and cisplatin were co-loaded onto ZIF67 via a co-incubation self-assembly approach to obtain the CCZ intermediate. Finally, FA-PEG was conjugated to the surface of CCZ to yield the final CCZF nanoreactor. Detailed synthesis parameters and optimization data are provided in the Supporting Information.

### 4.2 *In vitro* drug release assessment of CCZF nanoparticles

*In vitro* drug release from CCZF nanoparticles was evaluated using a dialysis method. Briefly, 2 mL of the nanoparticle suspension was transferred into a semipermeable dialysis membrane with a molecular weight cutoff (MWCO) of 8000–14000 Da. The dialysis apparatus was immersed in 20 mL of buffer solutions with varying H_2_O_2_ levels (0, 10, and 50 μM) within centrifuge vessels. The experimental setup was maintained under controlled conditions using an orbital shaker (100 rpm, 37°C, and pH = 6.8) throughout the investigation. At predetermined time points (0, 12, 24, 36, 48, 60, and 72 h), 1 mL aliquots were withdrawn from the external buffer solution. Following each extraction, an equivalent volume of fresh buffer solution was replenished to maintain constant volume. The quantification of released cisplatin (ICP-MS) and Co ions (spectrophotometric analysis) were performed, with cumulative release percentages calculated based on predetermined calibration curves; 25HC concentration was quantified via HPLC (mobile phase: methanol-water = 90 : 10, flow rate = 1 mL min^-1^, λ = 242 nm). The enzymatic kinetics of free CH25H and CCZF-encapsulated CH25H were evaluated using cholesterol as the substrate. Briefly, reaction systems containing equal amounts of active CH25H were incubated with different concentrations of cholesterol under identical experimental conditions. The substrate concentrations were set at 0, 10, 20, 40, 80, 120, 160, and 200 μM. After incubation for a fixed period within the initial linear reaction range, the product formation was quantified, and the initial reaction velocity (v0) was calculated. Each concentration was tested in triplicate. The kinetic parameters were determined by nonlinear fitting of the Michaelis–Menten equation:

$$v=\frac{V\;\max\;\left[S\right]}{Km+\left[S\right]}$$

where *v* is the initial reaction velocity and *[S]* is the substrate concentration. Lineweaver–Burk plots were additionally generated as supplementary linear transformations of the kinetic data.

### 4.3 Cellular uptake and efflux analysis of cisplatin

T24R cells were seeded into 6-well culture plates at a density of 1.5 × 10^5^ cells·mL^-1^ and allowed to adhere for 24 h. Cells were exposed to free cisplatin, Cisplatin@ZIF67 (CisZ), CCZ (CH25H@Cisplatin@ZIF67), and CCZF formulations, each adjusted to a final cisplatin concentration of 10 μg mL^-1^. Incubation periods were set at 0, 2, 4, and 8 h. Following treatment, cells were rinsed three times with PBS, fixed using 4% paraformaldehyde, and permeabilized with 0.1% Triton X-100. Cells were incubated with a cisplatin-specific primary antibody (1:200 dilution in 5% bovine serum albumin (BSA)) at 4°C overnight, followed by a phycoerythrin (PE)-conjugated secondary antibody (1:500 dilution) at room temperature for 1 h. For nuclear staining, 1 mL of DAPI solution (5 μg mL^-1^) was added for 15 min. After additional PBS washes, intracellular cisplatin-associated fluorescence (red) was visualized using confocal microscopy.

Intracellular cisplatin distribution was visualized by immunofluorescence staining with a cisplatin-DNA adduct-specific antibody as a semi-quantitative approach. For accurate determination of drug retention and efflux kinetics, absolute intracellular platinum content was quantified by ICP-MS as the gold-standard method. Flow cytometry with immunofluorescence staining was performed in parallel for intuitive comparison of relative intracellular drug levels. In a separate efflux study, T24R cells were cultured under identical conditions and treated with the same formulations at a cisplatin concentration of 10 μg mL^-1^ for 4 h. After removal of the drug-containing medium, the cells were replenished with fresh culture medium and incubated for varying durations (0, 1, 2, 4, 6, and 8 h). At each time point, the cells were harvested and subjected to flow cytometric analysis to evaluate drug retention and efflux dynamics.

T24R cells were treated with CCZF in the presence or absence of exogenous cholesterol supplementation. Soluble cholesterol was added to the culture medium at the indicated concentration for the designated duration. Cells were then collected for Western blot analysis of P-gp expression or for flow cytometric analysis of intracellular cisplatin retention/efflux. The cholesterol-only group received cholesterol supplementation without CCZF treatment.

### 4.4 Western blot quantification and reproducibility

Western blot experiments were performed using samples obtained from three independent biological replicates, and representative images are shown. Band intensities were quantified using ImageJ software (National Institutes of Health, Bethesda, MD, USA) and normalized to β-actin or GAPDH. For each experiment, the relative protein expression level was calculated by comparing the intensity of the target protein band with that of the corresponding β-actin or GAPDH band. Quantified data from three independent experiments were used for statistical analysis.

### 4.5 Assessment of cellular membrane fluidity

In this investigation, 1-pyrenedecanoic acid was employed as a fluorescent probe to evaluate the impact of CCZF nanoparticles on the fluidity of cell membranes. T24R cells were plated in 6-well culture dishes at a density of 2 × 10^5^ cells per well and allowed to adhere overnight. Following this, the cells were exposed to various treatments, including a control group, MβCD, CisZ, and CCZF (with a cisplatin concentration of 10 μg mL^-1^) for a duration of 2 h. After treatment, the cell suspensions were harvested and incubated with 2 μM 1-pyrenedecanoic acid for 5 min. Fluorescence measurements were then conducted using a spectrofluorometer. The membrane fluidity of each experimental group was determined by calculating the ratio of fluorescence intensity at 475 nm (corresponding to excimer formation) to that at 397 nm (representing monomer emission). This ratio served as an indicator of membrane fluidity, allowing for comparative analysis across the different treatment conditions.

### 4.6 Quantification of cellular cholesterol

T24R cells were seeded in 6-well plates at a density of 2 × 10^5^ cells per well and cultured overnight. Subsequently, the cells were treated with the following conditions: a control group, 5 mM MβCD, CisZ, and CCZF (cisplatin concentration: 10 μg mL^-1^) for 24 h. After treatment, the cell suspensions were collected, and cholesterol levels were measured using a commercially available cholesterol assay kit; 25HC concentration was quantified via HPLC (mobile phase: methanol-water = 90:10, flow rate = 1 mL min^-1^, λ = 242 nm).

### 4.7 Cell lines and animals

T24R cisplatin-resistant human bladder cancer cells were acquired from Jiangsu KeyGEN BioTECH Co., Ltd. (Nanjing, China). T24 human bladder cancer cells and L929 mouse fibroblasts were sourced from the Cell Bank of the Chinese Academy of Sciences (Shanghai, China). Murine bladder cancer MB49 cells were purchased from MeisenCTCC (Hangzhou, China). SV-HUC-1 human normal bladder epithelial cells were obtained from the American Type Culture Collection (ATCC). All cell lines were cultured in corresponding medium supplemented with 10% fetal bovine serum (FBS, Gibco) and 1% penicillin-streptomycin solution, and maintained in a humidified incubator at 37°C with 5% CO_2_. Specifically, T24/T24R and L929 cells used RPMI-1640 medium, and SV-HUC-1 cells used F-12K medium. T24R cells were continuously cultured with 2 μg mL^-1^ cisplatin to maintain the drug-resistant phenotype. The cisplatin-resistant murine bladder cancer cell line MB49R was established via stepwise dose escalation from the parental MB49 cell line. Briefly, parental MB49 cells were initially exposed to 0.5 μg mL^-1^ cisplatin, and the concentration was gradually increased over 3 months until cells stably proliferated in medium containing 2 μg mL^-1^ cisplatin. The resistance index was verified by MTT assay, with an IC_50_ value approximately 8.6-fold higher than that of parental MB49 cells. MB49R cells were maintained in RPMI-1640 medium containing 2 μg mL^-1^ cisplatin and passaged for at least 3 generations in drug-free medium before each experiment. Female C57BL/6 mice (6 weeks old) were obtained from SPF (Beijing) Laboratory Animal Technology Co., Ltd. All animal experimental protocols in this study were reviewed and formally approved by the Animal Ethics Committee of Qingdao University (Approval No. 20241015C574220241205133). All animal handling and experimental procedures were performed in strict compliance with the Guide for the Care and Use of Laboratory Animals published by the National Institutes of Health (NIH) and institutional ethical regulations for laboratory animal welfare. T24/T24R cells were used for in vitro mechanistic studies, whereas MB49R tumor-bearing C57BL/6 mice were used for *in vivo* antitumor efficacy and immune analyses.

### 4.8 *In vitro* STING inhibition assay using H-151

To verify the involvement of STING signaling in CCZF-induced immune activation, bladder cancer cells were pretreated with the STING inhibitor H-151 (1 μM) for 2 h and then incubated with CCZF for the indicated time. After treatment, total cellular proteins were extracted and subjected to western blot analysis to detect p-STING, p-TBK1, and p-IRF3. In parallel, the culture supernatants or cell lysates were collected for measuring IFN-β production by ELISA according to the manufacturer's instructions. The H-151-treated groups were used to evaluate whether CCZF-induced activation of the STING signaling cascade could be pharmacologically attenuated.

### 4.9 Establishment of MB49R tumor xenograft in mice

MB49R cells were harvested and suspended in a 1:1 (v/v) mixture of Matrigel matrix (Corning, USA) and PBS. A volume of 100 μL containing 1 × 10^7^ cells was injected subcutaneously into the right flank of female C57BL/6 mice. When the tumor volumes reached 60–100 mm^3^, the mice were selected for subsequent antitumor studies.

### 4.10 Tissue distribution study

Tumor-bearing C57BL/6 mice were randomly allocated into two groups, with five mice in each group. A volume of 100 μL of free IR783 or IR783@CCZF nanoparticles was administered through tail vein injection. Five tumor-bearing C57BL/6 mice were randomly divided into two groups: IR783 and IR783@CCZF. 24 h after injection, the major organs (heart, liver, spleen, lung, and kidney) along with tumors were excised. The fluorescence distribution in each tissue was captured and analyzed.

### 4.11 Evaluation of antitumor efficacy *in vivo*

All animal operations in this experiment were implemented in accordance with the ethically approved protocol (Approval No. 20241015C574220241205133). MB49R tumor-bearing C57BL/6 mice were randomly assigned to seven groups (n = 5 per group): Control, ZIF67, CZ, cisplatin, CisZ, CCZ, and CCZF (cisplatin dose: 10 mg kg^-1^). MB49R tumor-bearing C57BL/6 mice were randomly assigned to seven groups (n = 5 per group): Saline control, ZIF67, CZ, free cisplatin, CisZ, CCZ, and CCZF. All formulations were administered via tail vein injection at an equivalent cisplatin dose of 10 mg kg^-1^, with injections given every 3 days for a total of 4 treatments. Tumor volume and body weight were monitored every other day from day 1 to day 13, starting when tumors reached an initial volume of 60–100 mm^3^. Tumor volume was calculated using the formula: V = length × width^2^/2, where length and width represent the longest and shortest diameters of the tumor, respectively. At the end of the experiment, mice were sacrificed by cervical dislocation under anesthesia. Tumors and major organs (heart, liver, spleen, lungs, kidneys) were carefully harvested, weighed, and processed for subsequent tumor weighing, histological analysis, immunohistochemical staining, and biosafety evaluation.

### 4.12 Isolation and *In vitro* cultivation of BMDCs

BMDC were extracted from the femur and tibia of mice following a previously established protocol [55]. The isolated cells were filtered through a 70-μm mesh and treated with red blood cell (RBC) lysis buffer for 1 min. After centrifugation, the cells were resuspended in DMEM medium enriched with granulocyte-macrophage colony-stimulating factor (GM-CSF, 20 ng mL^-1^) and interleukin-4 (IL-4, 10 ng mL^-1^). To promote maturation, the supernatant from MB49R cells and various treatment conditions were sequentially introduced to the immature DC. The cell population, comprising both immature and mature DC, was stained with PE-conjugated anti-CD80 and FITC-conjugated anti-CD86 antibodies for 30 min. Following two washes, the samples were analyzed using a flow cytometer (BD Biosciences).

### 4.13 Long-term systemic toxicity assessment and ICP-MS analysis of cobalt biodistribution

Following the completion of treatment, mice were further observed for 30 days to evaluate the long-term biosafety of CCZF. During this period, body weight and general behavioral conditions were monitored. At the end of the observation period, blood samples were collected for serum biochemical analysis, including ALT, AST, UREA, and CREA. Major organs, including the heart, liver, spleen, lung, and kidney, were harvested, fixed in 4% paraformaldehyde, embedded in paraffin, sectioned, and stained with H&E for histopathological examination. To investigate the *in vivo* fate of cobalt after CCZF administration, residual cobalt levels in major organs were quantitatively determined by ICP-MS at different time points. At predetermined time points after administration, mice were sacrificed, and the heart, liver, spleen, lung, and kidney were collected, rinsed thoroughly with PBS, and weighed. The tissue samples were digested in concentrated nitric acid and diluted to a fixed volume with deionized water prior to ICP-MS analysis. The cobalt content was normalized to tissue weight and expressed as μg·g^-1^ tissue. For excretion analysis, urine samples were collected from mice at different time intervals after CCZF administration. The samples were appropriately diluted and analyzed by ICP-MS to quantify cobalt content, thereby evaluating the excretion kinetics of CCZF-derived cobalt.

### 4.14 Statistical analysis

All statistical analyses were performed using GraphPad Prism 8.0 software (GraphPad Software, La Jolla, CA, USA). All data are presented as mean ± standard deviation (SD), with n indicating the number of independent biological replicates. For comparisons between two groups, a two-tailed Student's t-test was used. For comparisons among three or more groups, a one-way analysis of variance (ANOVA) was performed, followed by Tukey's post-hoc test for multiple comparisons. Statistical significance was defined as follows: ns, not significant (P ≥ 0.05); *P < 0.05, **P < 0.01, ***P < 0.001, and ****P < 0.0001.

## Funding declaration

This study was financially supported by Taishan Scholar Program of Shandong Province (tstp20221165), the 10.13039/501100001809National Natural Science Foundation of China (82071750), and the Clinical Medicine + X Foundation of Affiliated Hospital of 10.13039/501100011324Qingdao University (QDFY + X2023137).

## CRediT authorship contribution statement

**Xianchun Fu:** Validation, Visualization, Writing – original draft, Writing – review & editing. **Ye Liang:** Resources, Software, Supervision. **Bin Xu:** Project administration, Resources, Software. **Chao Huang:** Investigation, Methodology, Project administration. **Pengfei Zhang:** Formal analysis, Investigation, Methodology, Project administration. **Haitao Niu:** Conceptualization, Data curation, Funding acquisition.

## Declaration of competing interest

The authors declare that they have no known competing financial interests or personal relationships that could have appeared to influence the work reported in this paper.

## Data Availability

Data will be made available on request.
