# Supplementary material for: A synergistic cascade nanoreactor breaks bladder cancer chemoresistance: Synergizing cholesterol metabolism reprogramming with cGAS-STING-mediated metallotherapy
Source: Mater Today Bio. 2026 Aug 6;40:103537. doi: 10.1016/j.mtbio.2026.103537 (PMC13487400; doi:10.1016/j.mtbio.2026.103537)
Supplement: Supplementary file 1 [file mmc1.docx]

**Supplementary Information for**

**A Synergistic Cascade Nanoreactor Breaks Bladder Cancer Chemoresistance: Synergizing Cholesterol Metabolism Reprogramming with cGAS-STING-Mediated Metallotherapy**

Xianchun Fu ^1, a^, Ye Liang ^1, a^, Bin Xu ^1, b^, Chao Huang ^c^, Pengfei Zhang ^a^, Haitao Niu ^a,^ ^*^

^a^ *Department of Urology,* *the Affiliated Hospital of Qingdao University, Qingdao 266003, China*

^b^ *Department of Hepatobiliary and Pancreatic Surgery, the Affiliated Hospital of Qingdao University, Qingdao 266003, China*

^c^ *College of Medicine, Southwest Jiaotong University, Chengdu 610031, Sichuan, China*

^1^ X. Fu, Y. Liang and B. Xu contributed equally to this work.

^*^ Corresponding authors.

*E-mail addresses*: niuht0532@126.com (H. Niu).

**Experimental section**

***Chemicals and Materials***

1. Core Reagents

Cobalt nitrate hexahydrate (99%, Sigma-Aldrich, Cat# 239267), 2-methylimidazole (99%, Sigma-Aldrich, Cat# M12609), folic acid-PEG-NHS (MW 5000, Nanocs, Cat# PG2-FANS-5k), cisplatin (99.9%, Sigma-Aldrich, Cat# P4394), methyl-β-cyclodextrin (MβCD, Sigma-Aldrich, Cat# C4555), STING inhibitor H-151 (MedChemExpress, Cat# HY-112710).

Cholesterol oxidase (BR, 10 U/mg), 2,2’-bipyridine-5,5’-dicarboxylic acid (GR), 3,3’,5,5’-tetramethylbenzidine dihydrochloride (purity > 99%) were sourced from Shanghai Macklin Biochemical Co., Ltd.

Europium-tetracycline (EUTC) was self-prepared for catalase activity detection.

RPMI-1640 medium, F-12K medium, penicillin-streptomycin solution and 0.25% trypsin-EDTA were purchased from KeyGEN BioTECH Co., Ltd.

Fetal bovine serum (FBS, Gibco, Cat# 10099-141) and Protein Ladder (Cat# 26616) were obtained from Thermo Fisher Scientific Inc.

Annexin V-FITC apoptosis detection kit was acquired from KeyGEN BioTECH Co., Ltd.

Total cholesterol assay kit was purchased from Nanjing Jiancheng Bioengineering Institute.

2. Recombinant Protein

Recombinant human cholesterol 25-hydroxylase (CH25H) was purchased from Novoprotein (Cat# P61329), with a specific activity ≥ 15 U/mg and purity ≥ 95% verified by SDS-PAGE.

3. Antibodies

Primary antibodies:

- Anti-P-glycoprotein (Abcam, Cat# ab170904, 1:1000 for Western blot)
- Anti-Flotillin-1 (Cell Signaling Technology, Cat# 18366, 1:1000 for Western blot)
- Anti-phospho-STING (Ser366, Cell Signaling Technology, Cat# 19782, 1:1000 for Western blot / 1:200 for IHC)
- Anti-HMGB1 (Abcam, Cat# ab79823, 1:500 for Western blot / 1:200 for immunofluorescence)
- Anti-Ki-67 (Abcam, Cat# ab15580, 1:200 for IHC)
- Anti-β-actin (Cell Signaling Technology, Cat# 3700, 1:5000 for Western blot)
- Anti-cisplatin (Abcam, Cat# ab103585, 1:200 for immunofluorescence / flow cytometry)

Secondary antibodies:

- HRP-conjugated goat anti-rabbit IgG (Beyotime, Cat# A0208, 1:5000 for Western blot)
- PE-conjugated goat anti-rabbit IgG (Beyotime, Cat# A0516, 1:500 for immunofluorescence / flow cytometry)

Flow cytometry immune antibodies (all from BioLegend, 1:200 working dilution):

Anti-CD45-FITC, anti-CD8-APC, anti-CD11c-PE, anti-MHC II-PerCP, anti-Foxp3-APC, anti-Gr-1-PE, anti-CD11b-FITC, anti-CD206-APC.

4. Instruments

Transmission electron microscopy (TEM) images were captured using an HT7700 microscope (Hitachi, Japan). Elemental mapping was performed on a Titan G2 60-300 transmission electron microscope (FEI, USA).

Fourier-transform infrared (FT-IR) spectra were recorded on a Nicolet iS10 spectrometer (Thermo Scientific, USA).

X-ray photoelectron spectroscopy (XPS) data were collected using a K-Alpha spectrometer (Thermo Scientific, USA).

UV-visible (UV-vis) spectra were measured with a UV-2600 spectrophotometer (Shimadzu, Japan).

Fluorescence imaging was conducted using Eclipse 80i (Nikon, Japan), A1R/A1 (Nikon, Japan), and TCS SP8 STED (Leica, Germany) microscopes.

Dissolved oxygen levels were quantified using an oxygen sensor (IPSJ 606l).

Flow cytometry data were acquired using a CytoFLEX S flow cytometer (Beckman Coulter).

Hypoxia experiments were performed in a hypoxic workstation (Ruskinn, UK).

***Cell Lines and Animals***

T24R cisplatin-resistant human bladder cancer cells were acquired from Jiangsu KeyGEN BioTECH Co., Ltd. (Nanjing, China).

T24 human bladder cancer cells and L929 mouse fibroblasts were sourced from the Cell Bank of the Chinese Academy of Sciences (Shanghai, China).

Murine bladder cancer MB49 cells were purchased from MeisenCTCC (Hangzhou, China).

SV-HUC-1 human normal bladder epithelial cells were obtained from the American Type Culture Collection (ATCC).

All cell lines were cultured in corresponding medium supplemented with 10% fetal bovine serum (FBS, Gibco) and 1% penicillin-streptomycin solution, and maintained in a humidified incubator at 37 °C with 5% CO₂. Specifically, T24/T24R and L929 cells used RPMI-1640 medium, and SV-HUC-1 cells used F-12K medium. T24R cells were continuously cultured with 2 μg·mL⁻¹ cisplatin to maintain the drug-resistant phenotype.

Female C57BL/6 mice (6 weeks old) were obtained from SPF (Beijing) Laboratory Animal Technology Co., Ltd. All procedures involving animals were conducted in compliance with the guidelines approved by the Animal Ethics Committee of Qingdao University, under the approval number 20241015C574220241205133.

***Synthesis of Metal-Organic Framework ZIF67***

ZIF67 was prepared following a modified procedure based on recent literature [1]. A modification step was included in the preparation of ZIF67. Initially, 224.6 mg (1.24 mmol) of 2-methylimidazole and 227 mg (1.67 mmol) of Co(NO_3_)_2_·6H_2_O were dissolved in 15 mL of methanol. The mixture underwent sonication for 3–5 minutes until complete dissolution of the solid, followed by magnetic stirring for 30 minutes. Subsequently, the solution was allowed to stand undisturbed for 12 hours at room temperature. After the standing period, the resulting product was centrifuged and washed three times alternately with ethanol and water at 8000 rpm. Finally, the product was vacuum dried at 60 °C for 24 hours.

***Preparation of CZ (CH25H@ZIF67) Nanoparticles***

4.5 mg of ZIF67 was dispersed in 4 mL of aqueous solution. 1.5 mg of CH25H was directly added to the above ZIF67 aqueous dispersion, and the mixture was subjected to a one-pot self-assembly reaction. The mixed system was incubated in a shaker at 37 °C overnight. The resulting CZ nanoparticles were collected by centrifugation (9000g, 5 minutes) and washed three times with PBS to remove unbound CH25H.

***Preparation of CCZF Nanoparticles***

5.0 mg of cisplatin was dissolved in 10 mL of PBS and then combined with 20 mg of CZ. The mixture was stirred at room temperature for 24 hours. The CCZ (Cisplatin@CH25H@ZIF67) nanoparticles were isolated by centrifugation (9000g, 5 minutes) and washed three times with PBS. CCZ nanoparticles were dispersed in a phosphate buffer solution, and the FA-PEG solution was added dropwise to the nanoparticle suspension under dark conditions. The mixture was stirred on a magnetic stirrer for 8 hours. Finally, the CCZF nano-system was obtained through ultrasonication.

***Dynamic colloidal stability assay***

CCZF nanoparticles were dispersed in 10% FBS, pH 6.8 PBS, or pH 6.8 PBS supplemented with 50 μM H₂O₂ and incubated at 37 °C. At predetermined time points (0, 6, 12, and 24 h), the hydrodynamic diameter, PDI, and zeta potential were measured by dynamic light scattering. Data are presented as mean ± SD (n = 3).

***CAT-Like Catalytic Activity of ZIF67 Nanoparticles***

A stock solution of 3-(N-morpholino) propane sulfonic acid (MOPS) buffer was prepared at 10 mM. In the assay, ZIF67 (200 μg·mL^-1^) was mixed with H_2_O_2_ (100 mM) and europium-tetracycline (EUTC, 0.126 mM) in the MOPS buffer, adjusting the total volume to 2 mL. Following a 10-minute incubation, fluorescence emission at 620 nm (excitation at 405 nm) was recorded. Additionally, dissolved oxygen levels were quantified using an oxygen sensor (IPSJ 606l). For another experiment, ZIF67 (final concentration 500 μg·mL^-1^) and H_2_O_2_ (final concentration 150 mM) were introduced sequentially into 3.0 mL of PBS. The amount of O_2_ produced (mg·L^-1^) was monitored at various time intervals.

***Enzyme activity assay before and after encapsulation***

To evaluate whether the loading process affected the enzymatic function of CH25H, the activities of free CH25H and CCZF-encapsulated CH25H were compared using cholesterol as the substrate. Equal amounts of CH25H, as determined by protein loading quantification, were incubated with 100 μM cholesterol at 37 °C for 10 min under normoxic conditions. The reaction time was selected based on preliminary time-course experiments to ensure measurement within the initial linear range. The generated 25-hydroxycholesterol was quantified by HPLC, and enzymatic activity was expressed as nmol product formed per min per mg CH25H. Relative activity was calculated by normalizing the activity of free CH25H to 100%. Blank CCZF was included as a negative control. All experiments were performed in triplicate.

***Enzyme activity assay under hypoxic conditions***

To assess the enzymatic activity of CH25H under hypoxia, free CH25H and CCZF-encapsulated CH25H were preincubated under normoxic conditions (21% O₂, 5% CO₂, balanced with N₂, 37 °C) or hypoxic conditions (1% O₂, 5% CO₂, 94% N₂, 37 °C) for 2 h in a hypoxic workstation. Subsequently, enzyme reactions were carried out in the corresponding atmosphere using 100 μM cholesterol as the substrate at 37 °C for 10 min. Product formation was quantified by HPLC, and the initial activity was calculated as described above. Relative activity was normalized to that of free CH25H under normoxia. All measurements were performed in triplicate.

***Real-time intratumoral oxygen measurement.***

MB49R tumor-bearing mice were used to evaluate intratumoral oxygen dynamics after different treatments. When the tumor volume reached approximately 100 mm³, mice were randomly divided into four groups and treated with PBS, free CH25H, CZ, or CCZF. At the indicated time point after administration, mice were anesthetized, and a needle-type oxygen microsensor was carefully inserted into the tumor at a consistent depth to monitor the intratumoral oxygen level in real time. Oxygen content was recorded at 0, 5, 10, 20, 30, 45, and 60 min, and the values were expressed in mmHg. To minimize measurement bias, the insertion position and depth of the microsensor were kept as consistent as possible for all animals. Data are presented as mean ± SD (n = 5).

***Hypoxia rescue assay for CH25H-mediated cholesterol oxidation***

To evaluate whether ZIF67-derived oxygen could preserve CH25H catalytic activity under hypoxic conditions, free CH25H, CZ, and CCZF were incubated with cholesterol substrate under normoxia (21% O₂) or hypoxia (1% O₂). For hypoxic experiments, the reaction system was pre-equilibrated in a hypoxia chamber before substrate addition. Briefly, each formulation was mixed with cholesterol-containing reaction buffer and incubated at 37 °C for 4 h under the indicated oxygen condition. After incubation, the residual cholesterol level was quantified using a commercial cholesterol assay kit according to the manufacturer’s instructions. Cholesterol degradation was calculated according to the following equation:

$$Cholesterol degradation \left( \% \right)=\frac{C_{0}-C_{t}}{C_{0}} \times100\%$$

Where $C_{0}$ and $C_{t}$ represent the cholesterol concentrations before and after reaction, respectively.

The relative activity retention under hypoxia was calculated based on cholesterol degradation as follows:

$$Relative activity retention (\%)=\frac{cholesterol degradation under hypoxia}{cholesterol degradation under normoxia} \times100\%$$

Data are presented as mean ± SD (n = 4).

***Free folate competition assay for cellular uptake***

To evaluate whether folate modification enhanced nanoparticle uptake through FRα-mediated recognition, coumarin-6-labeled CCZF and FA-PEG-CCZF nanoparticles were prepared and incubated with T24R cells. For the competition group, cells were pre-incubated with free folic acid (5 mM) for 1 h before addition of coumarin-6-labeled FA-PEG-CCZF. After incubation with the indicated formulations for 4 h, cells were washed three times with PBS, collected by trypsinization, and analyzed by flow cytometry. The intracellular fluorescence intensity was quantified as mean fluorescence intensity (MFI).

***Flow cytometry analysis of tumor and TDLN immune cells***

At 14 days post-treatment, tumor tissues and tumor-draining lymph nodes (TDLNs) were collected and processed into single-cell suspensions as described above. Before antibody staining, cells were incubated with anti-CD16/32 antibody for 10 min to block Fc receptors. Cells were then stained with Ghost viability dye and fluorophore-conjugated antibodies against CD45, CD3, CD4, CD8, F4/80, CD206, CD11b, Gr-1, CD11c, and MHC II. For intracellular Foxp3 staining, cells were fixed, permeabilized with 0.2% Triton X-100, and incubated with anti-Foxp3 antibody. For flow cytometric analysis, gating was performed sequentially as follows: (i) debris was excluded based on FSC-A versus SSC-A; (ii) single cells were selected using FSC-H versus FSC-A; (iii) live cells were identified as Ghost dye-negative events; and (iv) CD45^+^ leukocytes were used as the parent population for immune subset analysis. Immune cell subsets were defined as follows: CD8^+^ T cells, CD45^+^ CD3^+^ CD8+; Tregs, CD45^+^ CD3^+^ CD4^+^ Foxp3^+^; M2 macrophages, CD45^+^ F4/80^+^ CD206^+^; MDSCs, CD45^+^ CD11b^+^ Gr-1^+^; and mature DCs in TDLNs, CD45^+^ CD11c^+^ MHC II^+^. Gating boundaries were determined based on the corresponding controls and were applied consistently across all groups. Data were acquired using a CytoFLEX S flow cytometer (Beckman Coulter) and analyzed with the instrument-associated analysis software.”

***RNA sequencing and bioinformatics analysis***

Total RNA was extracted using TRIzol reagent. RNA integrity was evaluated using an Agilent 2100 Bioanalyzer. cDNA libraries were constructed and sequenced on the Illumina NovaSeq 6000 platform. Raw reads were trimmed by Trimmomatic and aligned to the human reference genome (GRCh38) using HISAT2. Gene expression levels were quantified by featureCounts. Differentially expressed genes were analyzed using DESeq2. Genes with padj < 0.05 and |log₂(fold change)| > 1 were considered significant DEGs. GO and KEGG enrichment analyses were performed using the R package *clusterProfiler*. Hypergeometric test with Benjamini–Hochberg correction was applied for enrichment analysis.

***Real-time intratumoral oxygen measurement***

MB49R tumor-bearing C57BL/6 mice were anesthetized and treated with PBS, ZIF67/CisZ/CCZF as indicated. Intratumoral oxygen content was monitored using a dissolved oxygen microsensor inserted into the tumor core at predetermined time points after administration. Oxygen levels were continuously recorded for 60 mins, and the data were expressed as mg/L or mmHg. Care was taken to maintain a constant depth and measurement position across groups.

***Hypoxia rescue assay for CH25H-mediated cholesterol oxidation***

To evaluate whether ZIF67-derived oxygen could rescue CH25H activity under hypoxic conditions, free CH25H and CCZF were incubated with cholesterol-containing reaction buffer under normoxia (21% O₂) or hypoxia (1% O₂) at 37 °C. After incubation for 60 mins, cholesterol consumption and 25HC production were quantified using HPLC. For hypoxic experiments, all reaction systems were pre-equilibrated in a hypoxia workstation before substrate addition

1. **Supplementary Figures:**

**Figure. S1.** Size distribution of ZIF67, CZ, and CCZF via DLS (n = 3); PDI (polydispersity index): ZIF67 = 0.13 ± 0.02, CZ = 0.14 ± 0.03, CCZF = 0.12 ± 0.02. Statistical analysis was performed by one-way ANOVA with Tukey’s post-hoc test. Data are expressed as mean ± SD (n = 3).

**Figure. S2.** XPS survey spectrum of ZIF67.

**Figure. S3.** XPS survey spectrum of C1s.

**Figure. S4.** XPS survey spectrum of N1s.

**Figure. S5.** XPS survey spectrum of Pt 4f.

**Figure. S6.** Isotherm linear plot of ZIF67.

**Figure. S7.** pore size distribution of ZIF67.

**Figure. S8.** The drug loading capacity (DLC, %) of CCZ nanoparticles was evaluated by varying the mass ratios between cisplatin and CZ nanoparticles (specifically, 1:1, 1:1.5, 1:2, and 1:2.5). Statistical analysis was performed by one-way ANOVA with Tukey’s post-hoc test. Data are expressed as mean ± SD (n = 3).

.

**Figure. S9.** The standard curve of BSA.

**Figure. S10.** The standard curve of cisplatin.

Figure **S11**. STEM and EDS elemental mapping of CCZF nanoparticles. (a) STEM image of a single CCZF nanoparticle. (b) Co elemental mapping. (c) Pt elemental mapping. (d) Overlay of Co and Pt mapping.

**Figure. S12.** Time-dependent changes in the hydrodynamic size and polydispersity index (PDI) of CCZF nanoparticles incubated in PBS for 7 days. Statistical analysis was performed by one-way ANOVA with Tukey’s post-hoc test. Data are expressed as mean ± SD (n = 3).

**Figure. S13.** Time-dependent changes in the hydrodynamic size and polydispersity index (PDI) of CCZF nanoparticles incubated in mouse serum for 24 h. Statistical analysis was performed by one-way ANOVA with Tukey’s post-hoc test. Data are expressed as mean ± SD (n = 3).

**Figure. S14.** Time-dependent changes in hydrodynamic diameter and polydispersity index (PDI) of CCZF nanoparticles incubated in 10% FBS, pH 6.8 PBS, and pH 6.8 PBS containing 50 μM H₂O₂ for 24 h. Statistical analysis was performed by one-way ANOVA with Tukey’s post-hoc test. Data are expressed as mean ± SD (n = 3).

**Figure. S15.** Time-dependent changes in zeta potential of CCZF nanoparticles incubated in 10% FBS, pH 6.8 PBS, and pH 6.8 PBS containing 50 μM H₂O₂ for 24 h. Statistical analysis was performed by one-way ANOVA with Tukey’s post-hoc test. Data are presented as mean ± SD (n = 3).

**Figure. S16.** Hemolysis test of CCZF nanoparticles. Statistical analysis was performed by one-way ANOVA with Tukey’s post-hoc test. Data are expressed as mean ± SD (n = 3).

**Figure. S17.** The cell viability of L929 cells incubated with ZIF67 (n = 3). Statistical analysis was performed by one-way ANOVA with Tukey’s post-hoc test. Error bars represent mean ± SD; ns, not significant.

**Figure. S18.** Changes in UV-vis absorption and color of ZIF-67 aqueous solutions at different H_2_O_2_ concentrations.

**Figure. S19.** Cumulative Co^2+^ release from CCZF under normal physiological conditions (pH 7.4) and TME-mimicking conditions (pH 5.5). Statistical analysis was performed by one-way ANOVA with Tukey’s post-hoc test. Data are presented as mean ± SD (n = 3).

**Figure. S20.** Cumulative cisplatin release from CCZF under normal physiological conditions (pH 7.4) and TME-mimicking conditions (pH 5.5). Statistical analysis was performed by one-way ANOVA with Tukey’s post-hoc test. Data are presented as mean ± SD (n = 3).

**Figure. S21.** The catalytic activity of ZIF67 toward H_2_O_2_ decomposition was monitored through fluorescence emission signals (λ_em_= 620 nm) generated by the EUTC.

**Figure. S22.** O_2_ evolution was observed during the ZIF67-catalyzed breakdown of H_2_O_2_.

**Figure. S23.** Time-dependent CAT-like activity curves of CCZF.

**Figure. S24.** The POD-like activity of ZIF67.

**Figure. S25.** Relative enzymatic activity of CH25H before and after encapsulation. Data are presented as mean ± SD (n = 3). Statistical significance was analyzed by one-way ANOVA. ns, not significant, *****P* < 0.0001.

**Figure. S26.** Enzymatic activity of free and CCZF-encapsulated CH25H under normoxic and hypoxic conditions. Data are presented as mean ± SD (n = 3). Statistical significance was analyzed by one-way ANOVA. ns, not significant, ****P* < 0.001, *****P* < 0.0001.

**Figure. S27.** Michaelis–Menten kinetic analysis of free CH25H and CCZF-encapsulated CH25H using cholesterol as the substrate. Statistical analysis was performed by one-way ANOVA with Tukey’s post-hoc test. Data are presented as mean ± SD from three independent experiments.

**Figure. S28.** Lineweaver–Burk plots of free CH25H and CCZF-encapsulated CH25H. Statistical analysis was performed by one-way ANOVA with Tukey’s post-hoc test. Data are presented as mean ± SD from three independent experiments.

**Table S1. Michaelis–Menten kinetic parameters of free CH25H and CCZF-encapsulated CH25H using cholesterol as the substrate.**

| **Sample** | **Vmax (nmol·min⁻¹·mg⁻¹)** | **Km (μM)** | **Relative Vmax retention** |
| --- | --- | --- | --- |
| Free CH25H | 105.2 ± 4.3 | 48.6 ± 3.1 | 100% |
| CCZF-encapsulated CH25H | 95.8 ± 3.9 | 61.4 ± 4.5 | 91.1% |

*Kinetic parameters were obtained by nonlinear fitting of the Michaelis–Menten equation. Statistical analysis was performed by one-way ANOVA with Tukey’s post-hoc test. Data are presented as mean ± SD (n = 3).*

**Figure. S29.** Real-time intratumoral oxygen monitoring after treatment with PBS, free CH25H, CZ, or CCZF in MB49R tumor-bearing mice. Data are presented as mean ± SD (n = 5). Statistical significance was analyzed by one-way ANOVA. ns, not significant, *****P* < 0.0001.

**Figure. S30.** Comparison of cholesterol degradation under normoxic and hypoxic conditions. Data are presented as mean ± SD (n = 4). Statistical significance was analyzed by one-way ANOVA. ns, not significant, *****P* < 0.0001.

**Figure. S31.** Relative activity retention of free CH25H, CZ, and CCZF under hypoxic conditions. Data are presented as mean ± SD (n = 4). Statistical significance was analyzed by one-way ANOVA. ns, not significant, ***P* < 0.01, *****P* < 0.0001.

**Figure. S32.** The time-dependence of CCZF on cholesterol metabolism (n = 4), Statistical analysis performed using one-way ANOVA. Error bars represent mean ± SD.

**Figure. S33.** The dose-dependence of CCZF on cholesterol metabolism (n = 4), Statistical analysis performed using one-way ANOVA. Error bars represent mean ± SD.

**Figure. S34.** Representative Western blot images showing P-gp protein expression in T24R cells treated with Control, Cholesterol only, CCZF, or CCZF + Cholesterol.

**Figure. S35.** Quantification of P-gp protein expression normalized to β-actin. Data are presented as mean ± SD from three independent experiments. Statistical significance was analyzed by one-way ANOVA. ns, not significant, *****P* < 0.0001.

**Figure. S36.** Representative flow cytometry histograms showing intracellular cisplatin retention in T24R cells from the Control, Cholesterol only, CCZF, and CCZF + Cholesterol groups. Cells were exposed to cisplatin, washed, and incubated in drug-free medium for the indicated period before intracellular cisplatin was detected with a cisplatin-specific antibody and a PE-conjugated secondary antibody.

**Figure. S37.** Quantitative analysis of intracellular cisplatin retention expressed as relative fluorescence intensity. Statistical analysis was performed by one-way ANOVA with Tukey’s post-hoc test. Data are presented as mean ± SD from three independent experiments. **P* < 0.05, ***P* < 0.01, ****P* < 0.001.

**Figure. S38.** The cell viability of T24R cells incubated with CCZF for 24 and 48 h (n = 4). Statistical analysis performed using one-way ANOVA. Error bars represent mean ± SD.

**Figure. S39.** Viability of T24R cells after 24 and 48 hours of cisplatin treatment (n = 4). Statistical analysis performed using one-way ANOVA. Error bars represent mean ± SD.

**Figure. S40.** The cell viability of T24 cells incubated with cisplatin for 24 and 48 h (n = 4). Statistical analysis performed using one-way ANOVA. Error bars represent mean ± SD.

**Figure. S41.** The cell viability of T24 cells incubated with different preparations for 24 and (n = 4). Statistical analysis performed using one-way ANOVA. Error bars represent mean ± SD, ns, not significant, *****P <* 0.0001.

**Figure. S42.** The cell viability of T24 cells incubated with different preparations for 48 h (n = 4). Statistical analysis performed using one-way ANOVA. Error bars represent mean ± SD, ns, not significant, *****P <* 0.0001.

**Figure. S43.** The cell viability of T24R cells incubated with different preparations for 24 h (n = 4), Statistical analysis performed using one-way ANOVA. Error bars represent mean ± SD; *****P <* 0.0001.

**Figure. S44.** Quantitative analysis of apoptosis rates in T24R cells after 24-hour treatment with different formulations (n = 4). Statistical analysis performed using one-way ANOVA. Error bars represent mean ± SD; ns, not significant, **P <* 0.05, ***P <* 0.01, *****P <* 0.0001.

**Figure. S45.** Cell viability of normal SV-HUC-1 cells assessed by MTT assay (n = 4). Statistical analysis performed using one-way ANOVA. Error bars represent mean ± SD; ns, not significant, *****P <* 0.0001.

**Figure. S46.** Changes in cytoplasmic mtDNA at different time points after CCZF treatment (n = 3). Statistical analysis performed using one-way ANOVA. Error bars represent mean ± SD; **P <* 0.05, ***P <* 0.01, ****P <* 0.001, *****P <* 0.0001.

**Figure. S47.** Changes in the expression of p-STING protein at different time points after CCZF treatment (n = 3). Statistical analysis performed using one-way ANOVA. Error bars represent mean ± SD; ****P <* 0.001, *****P <* 0.0001.

**Figure. S48.** Changes in IFN-β secretion at different time points after CCZF treatment (n = 3). Statistical analysis performed using one-way ANOVA. Error bars represent mean ± SD; ****P <* 0.001, *****P <* 0.0001.

**Figure. S49.** Representative western blot images showing the protein levels of p-STING, p-TBK1, p-IRF3 and GAPDH in T24R cells treated with Control, H-151, CCZF, or CCZF + H-151.

**Figure. S50.** Quantitative analysis of the relative phosphorylation levels of STING, TBK1, and IRF3 (n = 3). Statistical analysis performed using one-way ANOVA. Error bars represent mean ± SD; **P <* 0.05, ***P <* 0.01, ****P <* 0.001, ns, not significant.

**Figure. S51.** IFN-β production in bladder cancer cells after different treatments, as measured by ELISA. Data are presented as mean ± SD (n = 3). Statistical analysis performed using one-way ANOVA. *****P <* 0.0001.

**Figure. S52.** Quantitative analysis of total fluorescence intensity of HMGB1 in Control and CCZF groups (n = 3). Statistical analysis performed using one-way ANOVA. Error bars represent mean ± SD; *****P <* 0.0001.

**Figure. S53.** Schematic representation of BMDC isolation, culture, and analytical characterization.

**Figure S54**. Volcano plot of differentially expressed genes. RNA-seq was performed with three biological replicates in each group. Genes with padj < 0.05 and |log₂(fold change)| > 1 were defined as significantly differentially expressed. Red dots: significantly upregulated genes; blue dots: significantly downregulated genes; gray dots: non-significant genes. Key genes involved in cholesterol metabolism and immune activation are labeled.

**Figure S55**. Heatmap showing expression patterns of key genes. Relative expression levels of representative genes related to cholesterol metabolism and immune response. Gene expression was normalized by row-wise Z-score. Blue indicates low expression and red indicates high expression. Genes were clustered based on their expression profiles.

**Figure S56**. GO enrichment analysis of upregulated genes. GO enrichment analysis was performed for significantly upregulated genes. The size of each dot represents the number of enriched genes, and the dot color indicates adjusted *P* value. Representative enriched terms are shown.

**Figure S57**. GO enrichment analysis of downregulated genes. GO enrichment analysis was performed for significantly downregulated genes. The dot size corresponds to gene number, and the dot color represents adjusted *P* value. Top enriched functional terms are presented.

**Figure S58**. KEGG pathway enrichment analysis of upregulated genes. KEGG enrichment analysis of genes with significant upregulation. Dot size indicates the number of enriched genes, and color represents adjusted *P* value. Major enriched signaling pathways are displayed.

**Figure S59**. KEGG pathway enrichment analysis of downregulated genes. KEGG enrichment analysis of genes with significant downregulation. Dot size indicates gene count, and color corresponds to adjusted *P* value. The most enriched metabolic pathways are shown.

**Figure. S60.** Validation of FRα-mediated cellular uptake of FA-PEG-CCZF by free folate competition.

(A) Representative flow cytometry histograms of coumarin-6 fluorescence in T24R cells after incubation with CCZF, FA-PEG-CCZF, or FA-PEG-CCZF following pre-treatment with excess free folic acid (5 mM).

(B) Quantitative analysis of mean fluorescence intensity (MFI) in different groups. Data are presented as mean ± SD (n = 4). Statistical analysis was performed using one-way ANOVA. *****P* < 0.0001.

**Figure. S61.** Time-dependent residual cobalt levels in major organs, including the heart, liver, spleen, lung, and kidney, after administration, as determined by ICP-MS. Statistical analysis was performed by one-way ANOVA with Tukey’s post-hoc test. Data are presented as mean ± SD (n = 3). The cobalt content gradually decreased over time in all examined organs, indicating progressive clearance *in vivo*.

**Figure. S62.** Cobalt levels detected in urine at different time points after CCZF administration, as determined by ICP-MS. Statistical analysis was performed by one-way ANOVA with Tukey’s post-hoc test. Data are presented as mean ± SD (n = 3). The excretion kinetics confirmed the gradual elimination of CCZF-derived cobalt from the body.

**Figure. S63.** Changes in cisplatin concentration in serum and tumor tissue after administration (n = 4). Statistical analysis performed using one-way ANOVA. Error bars represent mean ± SD; *****P <* 0.0001.

**Table. S2.** Pharmacokinetic parameters of cisplatin in serum after administration of free cisplatin and CCZF, determined by non-compartmental analysis.

| **Parameter** | **Unit** | **Cisplatin** | **CCZF** |
| --- | --- | --- | --- |
| Serum Cmax | ng·mL^-1^ | 215.6 | 182.5 |
| Serum Tmax | h | 1 | 2 |
| Serum AUC_0–24_ h | ng·h·mL^-1^ | 1520.4 | 2527.8 |
| Tumor Cmax | ng·g^-1^ | 186.5 | 708.7 |
| Tumor Tmax | h | 24 | 24 |
| Tumor AUC_0–24_ h | ng·h·g^-1^ | 2685.5 | 11648.1 |
| Tumor/Serum AUC ratio | - | 1.77 | 4.61 |
| 24 hours Tumor/Serum concentration ratio | - | 21.44 | 15.68 |

**Figure. S64. Representative flow cytometry gating strategies for immune cell analysis in tumors and TDLNs from MB49R tumor-bearing C57BL/6 mice.**

Cells were first gated to exclude debris based on FSC-A versus SSC-A, followed by doublet discrimination using FSC-H versus FSC-A to obtain single cells. Live cells were then selected as Ghost dye-negative events. From the live-cell population, CD45^+^ leukocytes were gated for downstream immune subset analysis. CD8^+^ T cells were identified as CD45^+^ CD3^+^ CD8^+^ cells, and Tregs were identified as CD45^+^ CD3^+^ CD4^+^ Foxp3^+^ cells. MDSCs were defined as CD45^+^ CD11b^+^ Gr-1^+^ cells. M2 macrophages were identified as CD45^+^ F4/80^+^ CD206^+^ cells. Mature dendritic cells (DCs) in TDLNs were defined as CD45^+^ CD11c^+^ MHC II^+^ cells. The same gating strategy was consistently applied across all treatment groups.

**Figure. S65.** Quantification of mature DCs in TDLNs from MB49R tumor-bearing C57BL/6 mice after different treatments (n = 5).

**Figure. S66.** Quantification of mature DCs in TDLNs from MB49R tumor-bearing C57BL/6 mice after different treatments (n = 5). Statistical analysis performed using one-way ANOVA. Error bars represent mean ± SD, ns, not significant, *****P <* 0.0001.

**Figure. S67.** Representative flow cytometry plots of CD8^+^ T cells in tumors from MB49R tumor-bearing C57BL/6 mice (n = 5).

**Figure. S68.** Quantification of CD8^+^ T cells in tumors from MB49R tumor-bearing C57BL/6 mice (n = 5). Statistical analysis performed using one-way ANOVA. Error bars represent mean ± SD, ns, not significant, *****P <* 0.0001.

**Figure. S69.** Flow cytometry plots of M2 macrophages (CD206^+^ F4/80^+^) in tumor from MB49R tumor-bearing C57BL/6 mice (n = 5).

**Figure. S70.** Quantification of M2 macrophages (CD206^+^ F4/80^+^) in tumor tissues from MB49R tumor-bearing C57BL/6 mice (n = 5). Statistical analysis performed using one-way ANOVA. Error bars represent mean ± SD, ***P <* 0.01, *****P <* 0.0001.

**Figure. S71.** Flow cytometry plots of MDSCs (CD11b^+^ Gr1^+^) in tumor from MB49R tumor-bearing C57BL/6 mice (n = 5).

**Figure. S72.** Quantification of MDSCs (CD11b^+^ Gr1^+^) in tumor from MB49R tumor-bearing C57BL/6 mice (n = 5). Statistical analysis performed using one-way ANOVA. Error bars represent mean ± SD, ns, not significant, *****P <* 0.0001.

**Figure. S73.** Flow cytometry plots of Tregs (CD4^+^ Foxp3^+^) in tumor from MB49R tumor-bearing C57BL/6 mice (n = 5).

**Figure. S74.** Quantification of Tregs (CD4^+^ Foxp3^+^) in tumor from MB49R tumor-bearing C57BL/6 mice (n = 5). Statistical analysis performed using one-way ANOVA. Error bars represent mean ± SD, ns, not significant, *****P <* 0.0001.

**Figure. S75.** Changes in immune cell populations on days 3, 7, and 14 after CCZF treatment (n = 4). Statistical analysis performed using one-way ANOVA. Error bars represent mean ± SD, ns, not significant, *****P <* 0.0001.

**Figure. S76.** Histological evaluation of major organs using H&E staining.

**Figure. S77.** Blood biochemistry results for alanine aminotransferase (ALT), aspartate aminotransferase (AST), urea (UREA), and creatinine (CREA) in tumor-bearing mice, *****P <* 0.0001 vs. Saline group, ns, not significant. Statistical analysis was performed by one-way ANOVA with Tukey’s post-hoc test. Data are presented as mean ± SD (n = 5).

**Figure. S78.** Body weight changes of mice during the 30-day post-treatment observation period. Statistical analysis was performed by one-way ANOVA with Tukey’s post-hoc test. Data are presented as mean ± SD (n = 5). No significant body weight loss was observed in the CCZF-treated group, indicating favorable long-term tolerability.

**Figure. S79.** Serum biochemical analysis of ALT, AST, UREA, and CREA levels in mice after 30 days of post-treatment observation. Data are presented as mean ± SD (n = 5). No significant liver or kidney dysfunction was observed in the CCZF-treated group. Statistical analysis was performed using two-way ANOVA, ns, not significant.

**Figure. S80.** Representative H&E-stained images of major organs (heart, liver, spleen, lung, and kidney) collected from mice at 30 days after treatment. No evident tissue injury, necrosis, or inflammatory infiltration was observed in the CCZF-treated group. Scale bars: 200 μm.

[1] T.-M. Cheng, S.-C. Wang, P.-Y. Lee, C.-S. Hsu, M.-W. Chen, S. Yougbaré, H.-C. Chen, C. Kongvarhodom, L.-Y. Lin, Y.-F. Wu, Novel insights of structure evolution between ZIF and hydroxide via controlled doses of ammonium bifluoride and applications on battery supercapacitor hybrids, Journal of Energy Storage 68 (2023) 107709.
